# Simulating cardiac fluid dynamics in the human heart

**DOI:** 10.1093/pnasnexus/pgae392

**Published:** 2024-09-10

**Authors:** Marshall Davey, Charles Puelz, Simone Rossi, Margaret Anne Smith, David R Wells, Gregory M Sturgeon, W Paul Segars, John P Vavalle, Charles S Peskin, Boyce E Griffith

**Affiliations:** Curriculum in Bioinformatics and Computational Biology, University of North Carolina, Chapel Hill, NC 27599, USA; Department of Pediatrics-Cardiology, Baylor College of Medicine and Texas Children’s Hospital, Houston, TX 77030, USA; Department of Mathematics, University of Houston, Houston, TX 77204, USA; Department of Mathematics, University North Carolina, Chapel Hill, NC 27599, USA; Department of Mathematics, University North Carolina, Chapel Hill, NC 27599, USA; Department of Mathematics, University North Carolina, Chapel Hill, NC 27599, USA; Department of Radiology, Duke University Medical Center, Durham, NC 27705, USA; Department of Radiology, Duke University Medical Center, Durham, NC 27705, USA; Division of Cardiology, Department of Medicine, University of North Carolina School of Medicine, Chapel Hill, NC 27599, USA; Courant Institute of Mathematical Sciences, New York University, New York, NY 10012, USA; Department of Mathematics, University North Carolina, Chapel Hill, NC 27599, USA; Department of Biomedical Engineering, University of North Carolina, Chapel Hill, NC 27599, USA; Carolina Center for Interdisciplinary Applied Mathematics, University of North Carolina, Chapel Hill, NC 27599, USA; Computational Medicine Program, University of North Carolina School of Medicine, Chapel Hill, NC 27599, USA; McAllister Heart Institute, University of North Carolina School of Medicine, Chapel Hill, NC 27599, USA

**Keywords:** cardiac mechanics, cardiac fluid dynamics, heart valves, fluid–structure interaction, computational model

## Abstract

Cardiac fluid dynamics fundamentally involves interactions between complex blood flows and the structural deformations of the muscular heart walls and the thin valve leaflets. There has been longstanding scientific, engineering, and medical interest in creating mathematical models of the heart that capture, explain, and predict these fluid–structure interactions (FSIs). However, existing computational models that account for interactions among the blood, the actively contracting myocardium, and the valves are limited in their abilities to predict valve performance, capture fine-scale flow features, or use realistic descriptions of tissue biomechanics. Here we introduce and benchmark a comprehensive mathematical model of cardiac FSI in the human heart. A unique feature of our model is that it incorporates biomechanically detailed descriptions of all major cardiac structures that are calibrated using tensile tests of human tissue specimens to reflect the heart’s microstructure. Further, it is the first FSI model of the heart that provides anatomically and physiologically detailed representations of all four cardiac valves. We demonstrate that this integrative model generates physiologic dynamics, including realistic pressure–volume loops that automatically capture isovolumetric contraction and relaxation, and that its responses to changes in loading conditions are consistent with the Frank–Starling mechanism. These complex relationships emerge intrinsically from interactions within our comprehensive description of cardiac physiology. Such models can serve as tools for predicting the impacts of medical interventions. They also can provide platforms for mechanistic studies of cardiac pathophysiology and dysfunction, including congenital defects, cardiomyopathies, and heart failure, that are difficult or impossible to perform in patients.

**Significance Statement**

Predictive mathematical models of blood flow in the heart can simulate cardiac physiology, pathophysiology, and dysfunction along with responses to interventions. However, existing models of the heart are limited in their abilities to predict valve performance, use realistic descriptions of tissue biomechanics, or predict the response of the heart to changes in loading conditions. The integrative model of the human heart introduced herein aims to address these limitations. It generates pressure–volume loops, valvular pressure–flow relationships, and vortex formation times that are in excellent agreement with clinical and experimental data. The model also captures realistic changes in cardiac output in response to changing loading conditions. Critically, these physiologic aspects emerge inherently from mechanistic interactions within our comprehensive description of cardiac physiology.

## Introduction

The heart is the most dynamic organ in the body and has been a focus of scientific and medical inquiry for millennia. Studies of the human heart began with detailed descriptions of its gross anatomy and have evolved to encompass a diverse set of research questions and approaches (1). Current studies vary widely in both methodology and scale, from wet-lab experiments of cell function to analytic characterization of muscle fiber orientation (2, 3). Animal models were some of the first systems used to understand the heart as a dynamic system in vivo (4, 5), but they are limited by the invasive nature of the experimental measurements, which impact the observed dynamics, as well as by the differences in anatomy and physiology of other animals as compared to humans. Imaging and catheterization studies provide a means to study human cardiac function in vivo, but although technological advances continue to improve these approaches, they can be costly, are limited in the detail of their measurements, and can pose risks to human subjects. Further, whereas methods for measuring cardiac function can assess the present state of the heart, models are critical for predicting its future states following growth, remodeling, or clinical intervention. Indeed, predictive mathematical models that capture important hemodynamic features can serve as platforms for treatment planning and medical device design (6). Important examples include models for predicting coronary flow measures and the impact of interventions for congenital heart defects (7, 8). However, creating a comprehensive model of the heart to be used in treatment planning is difficult due to the complex mechanobiology of heart tissue and the incompleteness of available experimental and clinical data. This paper introduces a new holistic description of the heart that brings us significantly closer to this goal.

Different approaches have been used to create computational models that account for various aspects of heart function. Several groups have created heart models using large-deformation structural mechanics together with simplified descriptions of the blood that neglect spatial variations in pressure and velocity (9–12). These models have been used in many contexts, including assessing annuloplasty ring sizing for stresses and myocardial remodeling (13) and investigating the role of left ventricular assist devices in restoring left and right ventricular function in acute heart failure (14). Structural approaches are well suited to assessing mechanical stresses following intervention, but they cannot capture any resulting changes in cardiac flow dynamics, including transvalvular pressure gradients, intracardiac vortex formation, or chamber washout (15).

Computational fluid dynamics approaches also have been developed to model intracardiac flow fields in situations in which the motion of the heart wall can be prescribed. Because it is difficult or impossible to prescribe the dynamics of the heart valves, some models that prescribe wall motion have been augmented by fluid–structure interaction (FSI) models of the valves (16, 17). Such models have been used to investigate the flow dynamics generated by artificial heart valves, including studies on impacts of device orientation and comparisons between device designs (18, 19). This approach can enable high-fidelity simulations of intracardiac flow but omits the mechanics of the heart wall. Consequently, such models cannot readily account for situations in which the wall motion is unknown, or in which wall motion changes in response to a physiological perturbation or a medical intervention. For instance, the Frank–Starling mechanism is a well-characterized physiological response in which increases in diastolic filling result in increased cardiac output (20). Interventions that impact preload, afterload, or valvular pressure–flow relationships, including right heart interventions (21), medical therapies for pulmonary (22) or systemic (23) hypertension, or structural interventions for valve disease (24), all can change cardiac output. This motivates alternative approaches in which cardiac output is not predetermined but rather is an emergent prediction of the model.

FSI models of the whole heart can account for dynamic interactions between the blood, the actively contracting myocardium, and the thin cardiac valve leaflets. To our knowledge, the earliest complete model of cardiac FSI was developed by Peskin and McQueen (25, 26) using the immersed boundary method (27). Although their model captured the complex interactions of the heart muscle, valves, and blood, its anatomy was idealized, and it described the biomechanics of the heart using systems of one-dimensional elastic fibers that are challenging to calibrate to human data. Another major whole heart FSI model, the UT-Heart (28, 29), was initially developed at the University of Tokyo and uses the arbitrary Lagrangian–Eulerian (ALE) method (30). It has been deployed in a variety of contexts, including personalized studies of surgical intervention for congenital heart disease (28) and studies of cardiac resynchronization therapy (31). More recently, Viola et al. (32) introduced an FSI model of the whole heart based on a GPU-enhanced immersed boundary method (33). Their model describes cardiac mechanics using springs with stiffnesses that are informed by the anisotropic properties of cardiac tissues (33). In their approach, the myocardium is described as a transversely isotropic material with exclusively axial loading (33), which neglects well-characterized orthotropic mechanical properties of the myocardium (28, 34–36). Bucelli et al. (37) also developed an FSI model of the left heart based on the ALE method that includes a detailed description of the biomechanics of the myocardium, but which uses a resistive surface method for the valves that effectively treats the valves as diodes that open and close solely in response to transvalvular pressure differences. Together, these models represent some of the most physiologically comprehensive models of the heart to date. However, none include biomechanically detailed, three-dimensional descriptions of the cardiac valves. The utility of a model with three-dimensional valves is motivated by the concept that valve thickness controls leaflet bending and directly impacts valve dynamics. Further, valve thickness changes with age. Indeed, even in the absence of disease, the valve leaflets of adults aged 60 and above are approximately twice as thick as those aged 20 and below (38).

In this paper, we introduce and benchmark a new comprehensive FSI model of the human heart. The model anatomy is derived from cardiac computed tomography (CT) imaging and includes fully three-dimensional descriptions of all major cardiac structures, including the atria, ventricles, mitral and tricuspid valves and their chordae tendineae and papillary muscles, aortic and pulmonary valves, and great vessels. The biomechanical models for the heart and valves are parameterized using experimental tensile test data obtained exclusively from human tissue specimens (36, 39–42). Model-based approaches (43–45) consistent with earlier experimental work (46–51) describe the heart’s fiber architecture. FSI simulations use the immersed finite element/finite difference (IFED) version (52–55) of the immersed boundary method (27), which automatically handles contact between structures, including the valve leaflets (43, 56–58). Several recent methodological developments enabled this model, including modern tetrahedral mesh generation techniques (59) and stabilized nodal IFED methods (54, 55).

This study demonstrates that the dynamics that emerge from our heart model are in excellent agreement with benchmark physiological data. These include pressure–volume loops, valvular pressure–flow relationships, and vortex formation time (VFT) indices (60, 61). We also show that the model generates a physiological response to changes in preload that are consistent with prior clinical studies of the Frank–Starling mechanism (21). To our knowledge, this important physiological mechanism has not been considered in prior FSI simulation studies of the heart; however, capturing this response is essential for predicting the changes in cardiac performance that follow perturbations to preload or afterload, or interventions for valvular stenosis or regurgitation. Together, these results illustrate the model’s potential for mechanistically describing cardiac function in both health and disease.

## Results

### Modeling human cardiac anatomy and physiology

**Fig. 1.**
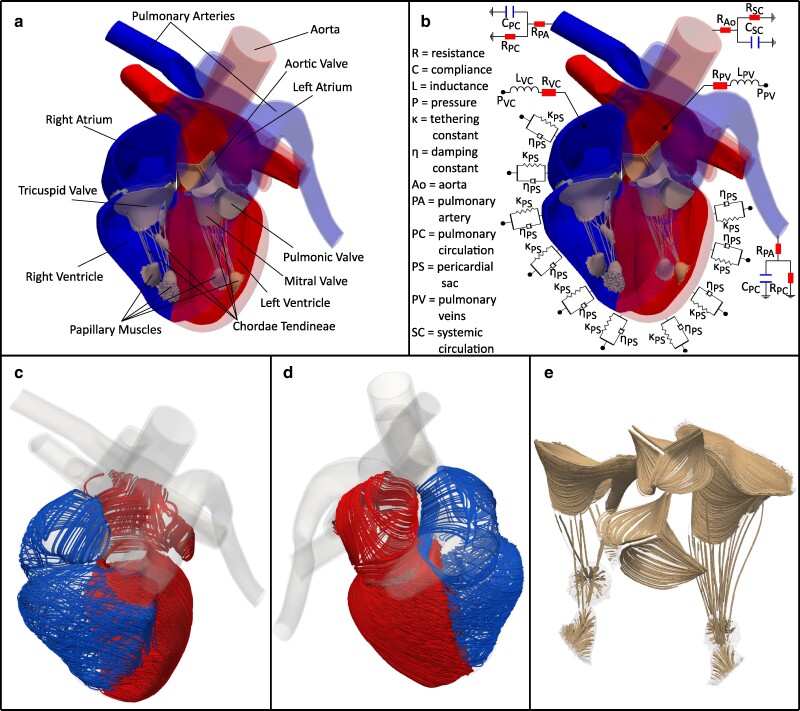
Anatomical and physiological aspects of the heart model. Panel a) visualizes the structural components of the heart model. The anterior portions of the chambers and great vessels are transparent to reveal the four valves and valvular complexes. Panel b) provides a schematic of the reduced-order models used for the pericardial sac, the systemic and pulmonary circulations, and the venous return to the atria. Panels c) and d) depict the main myocardial fibers from two different views with the right heart and left heart chambers depicted in blue and red, respectively, and panel e) visualizes the main fiber directions on the valve leaflets, chordae, and papillary muscles. The fiber coloration was chosen for visual clarity. The left heart appears on the right side of panels a–c, and it appears on the left side of panel d.

The defining feature of the present model is that all of its dynamics emerge from interactions among its components. We prescribe only the anatomy and physiology, including tissue properties, muscle activation timings, and physiological boundary conditions. Figure 1 provides an overview of the model, and Figure S1 shows the finite-element mesh used in our simulations.

The anatomy of the heart chambers and the nearby great vessels were reconstructed from deidentified cardiac CT images of a healthy adult male provided by Siemens Healthineers. The images used to reconstruct the model correspond to the early diastolic phase of the cardiac cycle, when the heart is in its most relaxed state (62). It can be difficult or impossible to capture the valve leaflets and chordae tendineae from whole heart CT images (63), and, indeed, the images used in this construction do not clearly show these structures. Consequently, we generated idealized anatomical models of the valve leaflets based on dimensions obtained from studies of human valves and merged these with the CT-derived chamber anatomy.

Cardiac tissues are highly anisotropic. In the myocardium, this a consequence of the alignment and organization of the muscle fibers (35). Likewise, the mechanical behavior of the valve leaflets is characterized by families of aligned collagen fibers (41). To capture these histological features within the modeled anatomy, we created a local coordinate system in each mesh element that is aligned with the principal or mean direction of anisotropy, such as the experimentally characterized relationships between fiber angle and transmural position within the ventricular myocardium (51). The mechanical responses of all structural components are defined by hyperelastic energy functionals, and the contractile mechanics of the myocardium are modeled by an active strain approach (64). Different strain-energy functionals are used for different structures to reflect their specific material properties, and different activation waveforms are specified for the atria and the ventricles. Supplementary Results Section *Left ventricular end-diastolic pressure-volume relationship* discusses our application of the methodology of Klotz et al. (65) to characterize the passive biomechanical response of the model left ventricle; see Figure S2.

In the body, a thin layer of pericardial fluid lubricates the interface between the myocardium and the pericardial sac, allowing the heart to slide along the pericardium as the heart contracts and relaxes. The pericardium constrains the motion of the ventricular wall, and accounting for these constraints is critical to achieve proper contraction and ventricular wall thickening during systole (66). As in earlier work (66), we model the effect of the pericardium on myocardial movement through a damped spring force applied in only the normal direction along the epicardial surface. These forces impose only a weak constraint on the chambers’ compliance relationships because they do not constrain the motion of the myocardium tangential to the pericardial surface.

At the length scale of the heart, blood behaves like a Newtonian fluid (25), and the dynamics of blood flow are well approximated by the incompressible Navier–Stokes equations. Afterload provided by the systemic and pulmonary circulations is described by Windkessel models (67) applied at the locations where the ascending aorta and the left and right pulmonary artery branches intersect the boundary of the computational domain. Venous return is modeled by pressure-driven flow sources located in each atrium (68). However, neither pressures nor flow rates are directly imposed at the connections to the arteries and veins. Instead, the model prescribes pressure–flow relationships. Depending on the relationships between the local pressures and flow rates in the detailed and reduced-order models, either inflow or outflow can be generated at any given connection, and the model neither assumes nor requires that the connections to the veins are always flow sources, nor that the connections to the arteries are flow sinks (68, 69).

We fit the model to achieve physiological blood pressures at physiological stroke volumes and heart rates. In the body, cardiac contractility and heart rate are actively controlled together with vascular tone to maintain systemic arterial blood pressure through a physiological control mechanism known as the baroreceptor reflex (70, 71). Vascular tone determines both the resistance and compliance of the blood vessels, and if it is held fixed, then increasing cardiac output acts to increase systemic blood pressure. Similarly, if cardiac output is held fixed, then modulating vascular tone can either raise or lower blood pressure; indeed, this is the physiological basis of many blood pressure medications. As detailed in Methods Section *Blood and circulation*, we use the ventricular output, as measured at the top of the ascending aorta, as feedback to produce physiological systemic arterial blood pressure ranges at a physiological stroke volume and heart rate to capture the action of the baroreceptor reflex.

We emphasize, however, that many of these benchmark metrics, such as cardiac output and stroke volume, vary widely across the adult population (72), and obtaining these statistics from both patients and healthy subjects often relies on indirect estimation (e.g. determining left ventricular volume as the volume of an ellipsoid with long axis and short axis measurements obtained via echocardiography). Because of the high variability of these performance statistics across the adult population, it is important to ensure that any cardiac model also captures other intrinsic qualitative features of the cardiac cycle. These include the pressure–volume relationships, such as the isovolumetric phases of the left ventricular pressure–volume loop, the shapes of the blood flow rate waveforms passing through the aortic and mitral valves, and the details of the intracardiac flow patterns. These dynamic relationships arise from the complex interplay between the chambers, the valves, and the circulatory system, were not calibrated in the model, and cannot be summarized by simple statistics.

### Chamber dynamics and pressure–volume relationships

**Fig. 2.**
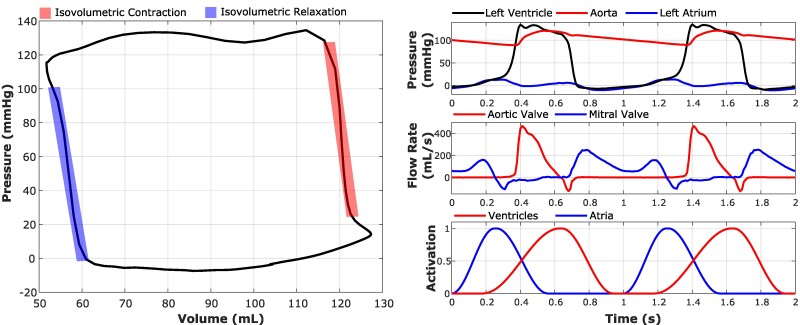
The left ventricular pressure–volume loop shown in the left panel captures characteristics of the cardiac cycle, including the isovolumetric phases and the stroke volume. The right panels show pressure, flow rate, and activation waveforms for two successive cardiac cycles.

Contraction in the heart model is driven using time-periodic atrial and ventricular activation waveforms at a heart rate of 60 beats per minute (BPM). The model reaches an approximate periodic steady state after five cycles. The results presented here focus on left heart dynamics because of the large body of available clinical and experimental data. The left ventricular pressure–volume relation, shown in Figure 2, characterizes ventricular performance. The left panel shows the pressure measured in the left ventricle plotted against the left ventricular volume to generate a pressure–volume loop. The right panels of Figure 2 depict pressure waveforms measured from the left atrium, left ventricle, and aorta as well as flow rate waveforms measured through the aortic and mitral valves. Supplementary Results Section *Left ventricular volume dynamics* details the left ventricular volume data, shown in Figure S3, that are used to generate the pressure–volume relation. Pressure–volume loop data are used to calculate end-diastolic volume (127.3 mL), end-systolic volume (51.73 mL), stroke volume (75.57 mL), ejection fraction (0.59), and cardiac output (4.5 L/min). These values can be compared to reference values for end-diastolic volume (110–120 mL) (70), end-systolic volume (40–50 mL) (70), stroke volume [70 mL (70) and (39.1–98.5 mL) (72)], ejection fraction (0.6) (70), and cardiac output [5 L/min (70) and (3.09–9.0 L/min) (72)]. These values are summarized in Table S5.

**Fig. 3.**
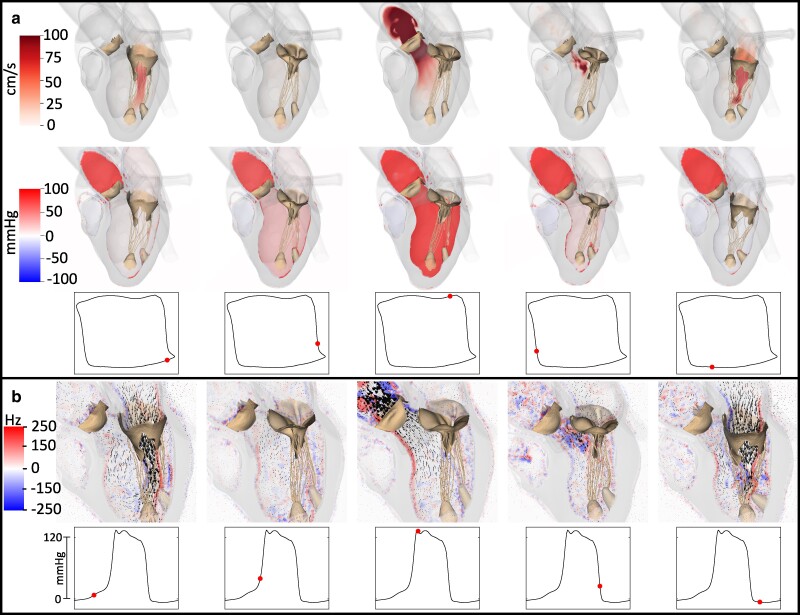
Cardiac fluid dynamics. a) Top and middle panels show renderings of blood velocity magnitude and pressure, respectively, along a plane bisecting the aortic and mitral valves with semi-translucent renderings of the chambers at five timepoints in the cardiac cycle. The bottom panels show the pressure–volume loop along with a red marker indicating the point in the cardiac cycle that is being visualized in the top and middle rows and share the axes scaling with the pressure–volume relation in Figure 2. See also Videos S1 and S2. b) Top panel shows renderings of blood velocity vectors along a plane bisecting the aortic and mitral valves with semi-translucent renderings of the chambers as well as the component of vorticity normal to the bisecting plane at five timepoints in the cardiac cycle. The left ventricular pressure waveform is included beneath, and the red marker corresponds to the point in the cardiac cycle that is being visualized in the top row. See also Video S3.

Figure 3a and Videos S1 and S2 show the blood velocity magnitude and pressure on a plane through the left atrium, left ventricle, and part of the ascending aorta that approximately bisects the aortic and mitral valves. These quantities are shown along with translucent renderings of the myocardium and fully opaque renderings of the aortic valve and the mitral valve apparatus. Each of the five columns corresponds to a timepoint in the cardiac cycle, including isovolumetric contraction (second column) and relaxation (fourth column). The red markers in the pressure–volume curves in the bottom row correspond to the same timepoints as the pressure and volume data in the top and middle rows. Figure 3b and Video S3 depict the planar vorticity and velocity vector field on a slice through the left heart with a time series plot of left ventricular pressure included beneath. Periods of isovolumetric contraction and relaxation are clearly seen in the second and fourth columns, respectively.

**Fig. 4.**
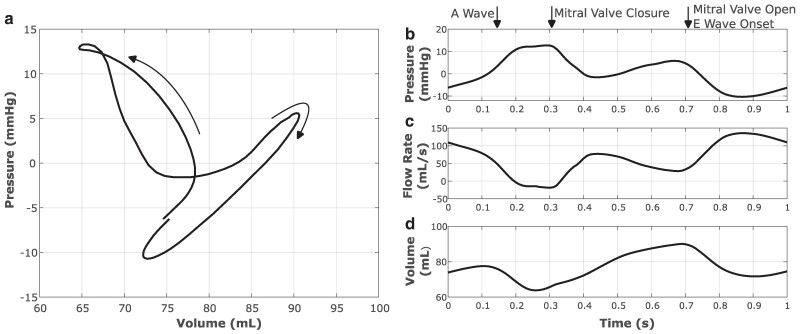
Quantification of the left atrial pressure–volume relation a), the time-series pressure data b), the source flow rate data c), and the time-series volume data d). The atrial pressure–volume loop (a) shows the quintessential “A loop” (left) and “V loop” (right) that clearly distinguish the atrial pressure–volume relation from the ventricular pressure–volume relation (73). Labels have been added to show the status of the mitral valve for the time-dependent data.

Pressure–volume relationships also characterize left atrial performance, and the difference between atrial and ventricular pressure–volume loops is apparent. Unlike the single loop of the ventricular pressure–volume relationship, the atrial pressure–volume relationship includes two lobes in a figure-eight pattern (73, 74), as shown in Figure 4a. This characteristic shape occurs because there are no structures to prevent retrograde flow to the veins. The A loop, which is on the left of Figure 4a, corresponds to atrial contraction and is traversed counterclockwise, and the V loop, which is on the right of Figure 4a, corresponds to passive filling and is traversed clockwise. The total volume change of the pressure–volume relation is called the atrial reservoir volume because the atrium is acting as a receptacle for blood from right ventricular contraction (75). The volume ejected from the atrium is split into two phases that approximately correspond to the flows associated with the A and V loops (73, 75). The volume from the active contraction is known as the pump volume, and this volume spans the A loop and is ejected from the atrium during the A wave in the mitral flow rate waveform. The pump volume is the difference between the maximum volume before peak contraction, which occurs at approximately 0.1 s in Figure 4b, and the minimum atrial volume. The initial volume ejected from the atrium into the ventricle, which occurs when the ventricle is relaxing, is the conduit volume. It is associated with the V loop and the E wave in the mitral flow rate waveform. The conduit volume is the difference between the ventricular filling volume and the combined reservoir and pump volumes (75). Backflow into the pulmonary veins is physiological and corresponds to the periods of negative flow seen in Figure 4c. Figure 4d shows the resulting atrial volume dynamics. We report the atrial reservoir, conduit, and pump volumes as fractions of the stroke volume as 0.344, 0.474, and 0.181, respectively, with reference values (73) of 0.375, 0.365, and 0.26, respectively. These values are summarized in Table S6.

### Cardiac valve dynamics

**Fig. 5.**
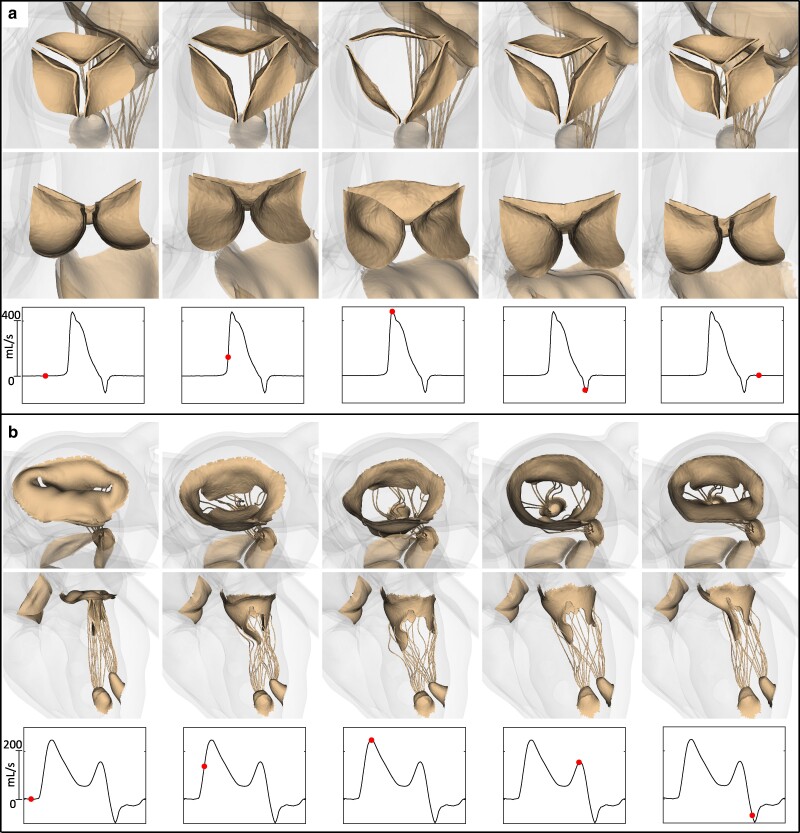
Cardiac valve dynamics. a) The top panels show the aortic valve deformations from two different views for five timepoints in the cardiac cycle. The bottom panels depict the aortic flow rate waveform along with a red marker corresponding to the same timepoint visualized in the cardiac cycle. See also Video S4. b) The top panels show the mitral valve deformations from two different views for five timepoints throughout a cardiac cycle. The bottom panels depict the mitral flow rate waveform along with a red marker corresponding to the same timepoint visualized in the cardiac cycle. See also Video S5.

Fully three-dimensional and biomechanically detailed descriptions of the cardiac valves are key characteristics of the model. Figure 5 and Videos S4 and S5 provide visualizations of the deformations of the aortic and mitral valves along with flow rate waveforms measured within the valve annuli. Both valves are shown from top and side views to highlight the opening and closing dynamics of the leaflets. The side view of the mitral valve includes the chordae and papillary muscles to highlight their role in maintaining closure of the valve during peak ventricular systole.

### Comparisons to clinical and experimental data

**Fig. 6.**
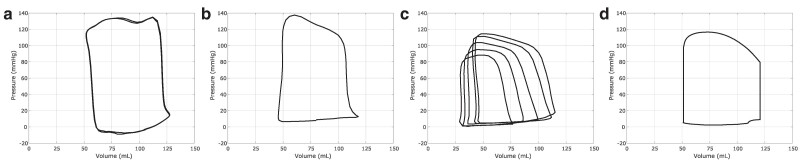
Successive pressure–volume curves from the fifth and sixth cycles of the model a) compared to clinical pressure–volume relations from a single cycle (digitized from Patterson et al. (76)) b), a Frank–Starling mechanism study via vena cava occlusion (digitized from Bastos et al. (21)) c), and a medical physiology textbook (digitized from Guyton & Hall (70)) d).

Figure 6 compares the left ventricular pressure–volume loops generated over successive cycles of the model to clinical pressure–volume relations (21, 76) and an example from a medical textbook (70). The effects of the mitral closing transients are apparent in the cusps seen in the bottom right corners of the clinical pressure–volume relations as in the model. In contrast, the aortic valve closure transients have a less noticeable impact on the upper left corners of the pressure–volume relation. Textbook pressure–volume loops generally show sharp transitions in and out of the isovolumetric phases (70), but clinical pressure–volume relations demonstrate that these transitions are not sharp and that the isovolumetric phases are not strictly volume preserving; see Figure 6d (70).

**Fig. 7.**
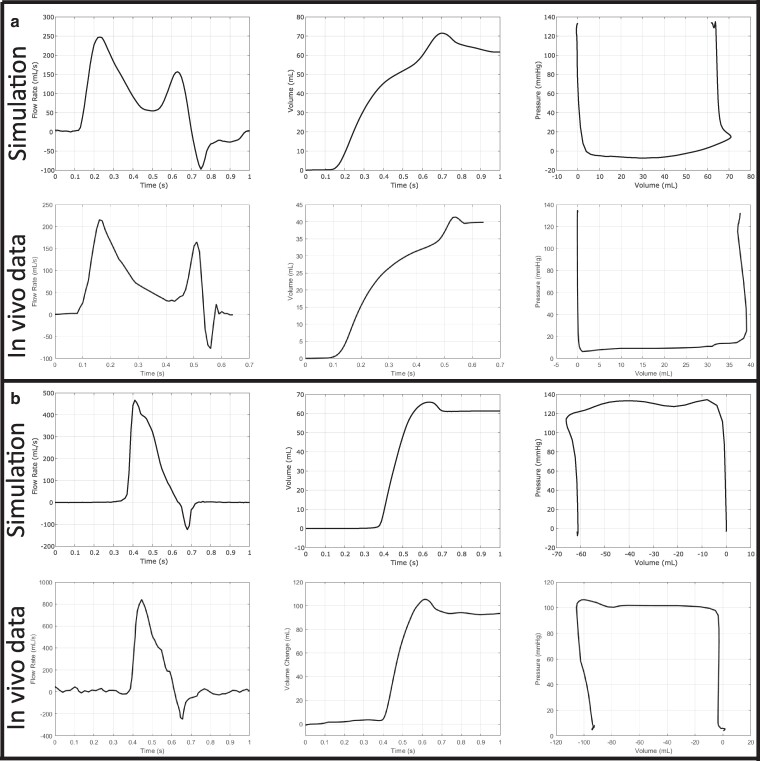
a) Comparison of in vivo canine mitral valve flow rate data (digitized from Yellin et al. (77)) to the model. The second column shows the volume that has passed through the mitral valve to the left ventricle during the cardiac cycle calculated by integrating the flow rate waveform. The third column shows the mitral valve contribution to the left ventricular pressure–volume relation. b) Comparison of in vivo human aortic valve flow rate data (digitized from Murgo et al. (78)) to the model. The second column shows the volume that has passed through the aortic valve into the aorta during the cardiac cycle calculated by integrating the flow rate waveform. The third column shows the aortic valve contribution to the left ventricular pressure–volume relation.

Figure 7a compares in vivo canine mitral flow rate data (77) to flow rate waveforms generated by the model. In the first column, it is apparent that the mitral flow rate waveform shape generated by the model is qualitatively similar to the in vivo data, including the mitral valve closing transient indicated by the negative flow rate. For the simulated and experimental flow rate waveforms, we compute the volume passing through the mitral valve over one cycle by integrating the flow rate data. The in vivo and model data look very similar in that there is a large increase in the volume output followed by a small loss in volume during the closing transient, which is more pronounced and prolonged in the model, followed by an eventual leveling of the volume. The right column plots the left ventricular pressure against the volume passing through the mitral valve to show the contribution of the mitral valve flux to the total pressure–volume relationship. The in vivo pressure data come from the same source as the flow rate data, and the flow rate and pressure were measured simultaneously (77). The simulation and experimental data show that the isovolumetric contraction periods are not truly isovolumetric because of the mitral valve closing transient and the method used to determine volume, which does not account for the dynamic fluid volume captured between the mitral valve ring and the closed mitral valve leaflets. These results are consistent with the clinical pressure–volume loops detailed in Figure 6.

Figure 7b compares digitized in vivo human aortic flow rate data (78) to data generated by the model. In the first column, it is apparent that the aortic flow rate waveform shape generated by the model is qualitatively similar to the clinical data, including the aortic valve closing transient indicated by the negative flow rate. For both cases, we compute the volume passing through the aortic valve over one cycle by integrating the flow rate. The clinical and simulated waveforms look very similar in that there is a large increase in the volume output followed by a small loss in volume during the closing transient and a leveling of the volume, as seen in the second column. The right column plots the left ventricular pressure against the volume passing through the aortic valve to show the contribution of the aortic valve flux to the total pressure–volume relationship, which shows clear volume gain during “isovolumetric” relaxation for both the model and in vivo data. The in vivo pressure data come from the same source as the flow rate data, and the flow rate and pressure were measured simultaneously (78).

**Fig. 8.**
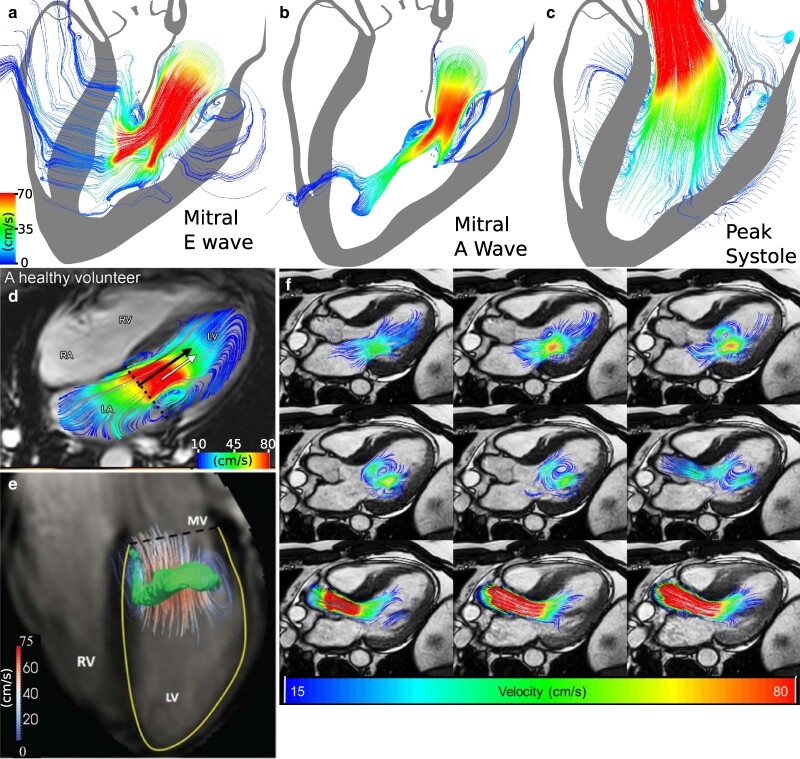
Comparison of instantaneous flow patterns generated by the model to in vivo data. Instantaneous streamlines are shown along two-dimensional slices that bisect the mitral valve and left ventricular outflow tract during peak E wave a), A wave b), and systole c). Notice that instantaneous streamlines passing through the heart wall are physical and reflect the wall motion. d) Streamlines generated from 4D flow MRI of a healthy subject during the E wave (adapted from Calkoen et al. (79)). e) Streamlines generated from 4D flow MRI of a healthy subject during the E wave with a superimposed vortex ring generated from $\lambda_{2}$-based methods (adapted from Elbaz et al. (61)). f) Streamlines generated from 4D flow MRI of a healthy subject from A wave onset (top left) to systolic ejection (bottom right) (adapted from Geest et al. (80)).

Streamlines help to characterize flow features that emerge during the cycle as a result of ejection through the mitral annulus. Figure 8 compares streamlines generated by the model to flow patterns from in vivo four-dimensinoal flow magnetic resonance imaging (4D flow MRI) data during the E- and A-waves and systolic ejection. We see reasonable qualitative agreement between the simulation and the in vivo streamline data during the E-wave (Figure 8a,d–e), with streamlines curling around the tips of the mitral leaflets. These flow features are an important contributor to vortex formation in the ventricle (61). The same behavior is noted during the A-wave in the model and in vivo, as seen in Figures 8b and f, respectively. Figure 8c illustrates ventricular ejection and shows that there is substantial flow throughout the ventricle, from apex to base. Although similarities in flow features indicate appropriate atrial and ventricular ejection dynamics, it is important to note that there are differences in methodologies used to capture these velocity fields. The velocity fields used to generate the streamlines in the model are taken at snapshots of single timepoints during the cycle, whereas 4D flow MRI relies on regenerating timepoints using composite images over multiple beats taken at approximately the same time in the cycle (80). This phase-averaging process will suppress fluctuations that are apparent in instantaneous flow field data.

To provide a quantitative assessment of the left ventricular flow field, we use an index proposed by Gharib et al. (81) called vortex formation time (VFT) to quantify vortex formation in the left ventricle during diastole. This index relies on readily available left ventricular benchmark metrics. It is defined as $\text{VFT}=\frac{4(1-\beta )}{\pi }\;\alpha^{3}\;\text{EF}$ for $\alpha =\frac{{\text{EDV}}^{1/3}}{\bar{D}}$, in which *β* is the fraction of stroke volume from atrial contraction, EF is the ejection fraction, EDV is the end-diastolic volume, and $\bar{D}$ is the mitral valve annulus diameter averaged over atrial ejection. This nondimensional value has been shown to be an effective clinical indicator of dilated cardiomyopathy (81) and heart failure diagnosis and progression (60). VFT is sensitive to the method used to determine mitral valve diameter (60, 82). In prior clinical studies on healthy human subjects, Poh et al. (60) identify a VFT of 2.67±0.8, and Elbaz et al. (61) identify a VFT of 2.6±0.6. Following the approach of Poh et al. (60), we find that the VFT generated by the model is 2.56, which is in excellent agreement with these in vivo findings.

### Physiological response to changes in loading: the Frank–Starling mechanism

An important advantage of an FSI model is its ability to predict changes in heart function that result from physiological changes in loading conditions, or that follow from medical therapies or structural interventions. For instance, cardiac preload can change in response to increased loads from epinephrine release during stress, or as a response to increased cavity pressures during the natural breathing cycle (83). The Frank–Starling mechanism is a cellular level beat-to-beat adaptation that helps modulate stroke volume to match incoming ventricular preload (83–86). We examine whether our model captures the Frank–Starling mechanism by simulating in vivo experiments in which increased end-diastolic volume leads to increased stroke volume (20, 21, 87).

**Fig. 9.**
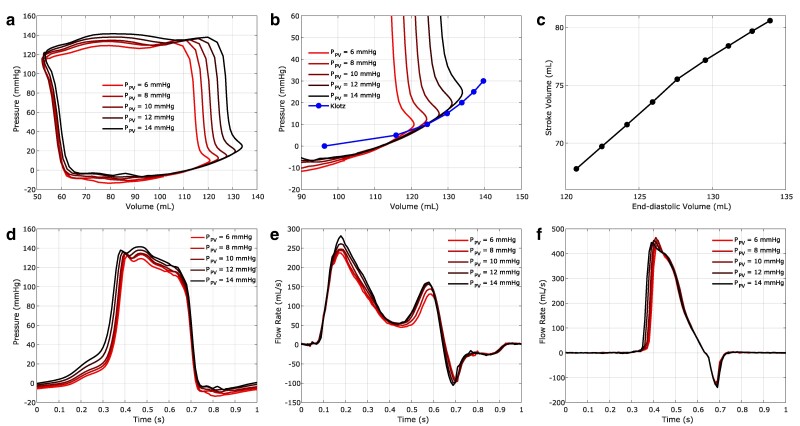
Frank–Starling mechanism in the model heart. a) Left ventricular pressure–volume relations across multiple cardiac cycles under changing pulmonary venous pressures. b) Comparison of the diastolic filling portion of the left ventricular pressure–volume loop to the static end-diastolic pressure–volume relationship analyzed in Figure S2. c) The left ventricular function curve, characterizes the relationship between left ventricular end-diastolic volume and resultant stroke volume. Changes in d) left ventricular pressure, e) mitral flow, and f) aortic flow that result from changes in pulmonary venous pressures that establish preload on the left heart.

Here we model human experiments that occlude the vena cava and then incrementally release the occlusion over successive cardiac cycles (20, 21, 87), which modulates the pulmonary venous pressures that establish preload for the left heart. We capture this effect in the model by changing the pulmonary venous pressure upstream of the left atrial flow source. First, the model is brought to a steady state over five cycles using a nominal pulmonary venous pressure load of 10 mmHg. Then, we perform two sets of simulations in which we systematically increase or decrease the pulmonary venous pressure, modulating the loading pressure by 1 mmHg in subsequent cycles to span pressures ranging from 6 to 15 mmHg. Figure 9a shows the resulting pressure–volume relationships. Figure 9b highlights differences between the dynamic end-diastolic pressure–volume relationship and the static relationship used in the Klotz curve analysis. Figure 9c shows the left ventricular function curve generated by the model, which indicates that increased filling generates increased ejection. Finally, Figure 9d–f show the ventricular pressure, mitral flow, and aortic flow waveforms that are generated by the model in response to changes in preload.

## Discussion

### Chamber dynamics and pressure–volume relationships

Pressure–volume relationships identify important quantitative and qualitative features of the cardiac cycle. These include the isovolumetric periods during contraction and relaxation, when pressure is respectively increasing and decreasing in the left ventricle, but neither the aortic valve nor the mitral valve are permitting flow. As highlighted in Figure 6, clinical measurements (21, 76) show that during the isovolumetric phases of the cardiac cycle, the volume is not strictly constant, in contrast with schematics presented in medical textbooks (70) and pressure–volume relations derived from simulations using ideal (diode-like) valve models (88). The lack of strict “isovolumetric” phases appears to be a result of the closing transients associated with both the mitral valve (Figure 7a) and aortic valve (Figure 7b), and our model reflects this observed in vivo behavior; see Figure 6. The characteristic shape of the mitral closing transient in the pressure–volume relation is not consistent across in vivo studies, and although our model reflects the initial sharp volume drop seen in Figure 6b,c and Figure 7b indicate a more gradual volumetric drop during isovolumetric contraction. Further, whereas other approaches to cardiac FSI require specialized numerical methods and additional physiological models to capture the isovolumetric phases of the cardiac cycle (89), a notable feature of our model is that it automatically captures the isovolumetric phases of the cardiac cycle.

The left atrial pressure–volume relationship shown in Figure 4a provides further insight into the function of both the atrium and ventricle. In normal heart function, the reservoir volume composes the largest percentage with respect to the ventricular filling volume, followed by the conduit volume and then pump volume (73). In the model, the conduit volume exceeds the ventricular filling volume, which suggests restrictive ventricular filling (73, 75). However, increased conduit volume is not necessarily pathological and is also found during intense physical exertion (75). Because other metrics of ventricular function do not indicate restrictive filling, these model outputs suggest that the atrial contraction may need to be increased. Atrial contraction dynamics generated by the model are also impacted by the presence of flow sources in the atrial chambers, but extensions of the model are needed to eliminate the need for these flow sources. Nonetheless, the left atrial A- and V-wave pressures, respectively 13.29 and 6.59 mmHg, are in the physiological range (90). Further, the overall left atrial pressure–volume loop is physiological and captures the key features of in vivo pressure–volume relationships (73).

### Valvular dynamics, pressure–flow relationships, and comparisons to in vivo data

Although some models use descriptions of the valves that open or close solely in response to inflow or outflow boundary conditions, in our model, the motion of the valve cusps is determined by fluid-structure interactions between the leaflets and the local blood flow. The thin valve leaflets move with the local blood velocity but, at the same time, apply forces to the blood that affect its motion. Consequently, interactions among all of the components of the heart along with preload and afterload determine the valvular dynamics along with their resulting pressure–flow relationships.

The aortic valve closure transient seen in column 4 of Figure 5a yields a small (4.01 mL) regurgitant flow volume and is in excellent qualitative agreement with human clinical data (78), as detailed in Figure 7b. The mitral flow rate waveform can be quantitatively assessed using the measured deceleration time and the ratio of the peak magnitudes of the E and A flow rate waveforms, which for our model are respectively 250 ms and 1.57. These values fall within the expected ranges for healthy adults (91). We remark, however, that clinical measurements typically use blood velocity waveforms obtained via Doppler echocardiography. Obtaining comparable measurements from the model would require simulating the acquisition protocols used clinically. The mitral valve flow rate waveform also captures many complex qualitative features of in vivo canine studies of mitral valve flow rates (77). These include a mitral valve closing transient during early ventricular contraction before the valve closes completely, highlighted in the fifth column of Figure 5b. During this period in our model, a small (5.27 mL) regurgitant flow volume is lost from the left ventricle into the left atrium. This behavior has been clearly seen in canine experimental studies, and Figure 7a provides a direct comparison between our model and such experimental data. Figure 8 shows that the ventricular flow patterns generated by the model are in qualitative agreement with in vivo flow patterns in healthy human hearts obtained using 4D flow MRI. In addition, the left ventricular vortex formation time (81) generated by our model (2.56) is in agreement with prior in vivo studies in healthy adult subjects (2.67±0.8 (60)), which provides a quantitative indication that the model is generating both flows and chamber dynamics, including motions of the mitral annulus, that are consistent with those of a healthy heart.

### Mechanical characterization

As detailed in Methods Section *Fiber architecture*, this study uses a model-based approach to describe the muscle fiber architecture of the heart and the collagen fiber architectures of the cardiac valves. Different methods have been used in prior work, including machine-learning methods that map fiber architectures obtained from histology studies onto patient- or subject-specific anatomies using geometric landmarks (92) and fiber structures determined through diffusion tensor MRI (93). Our modeling framework does not require the use of a unified approach to capture the influence of the tissue microarchitecture in all regions of the heart. Indeed, specializations of the model could use different approaches in different subregions that are appropriate for the data that are available for model calibration.

All of the constitutive models used in this study to describe the mechanical properties of the heart are parameterized from tensile tests of human tissue specimens (36, 39–42), which is common practice for tissue characterization in the cardiac modeling field (11, 32, 37, 94). Our approach, however, permits the use of alternative methods for material characterization. For instance, methods for inferring subject-specific tissue properties from noninvasive image data are being actively developed for in vivo parameter generation (95), but current estimations rely on indirect measures of stress and strain and assume the absence of residual stresses in early diastole. While these imaging modalities have been shown to be effective in patient-specific cases, the impact of using these parameters compared to tensile test parameters on models of cardiac FSI is unknown, and the parameters are not generalizable to alternate geometries.

To ensure the literature parameters describe proper passive material behavior of the left ventricle, we have recreated the Klotz curve (65) for comparison with clinical data. Supplementary Results Section *Left ventricular end-diastolic pressure-volume relationship* shows the Klotz curve, which characterizes the left ventricular end-diastolic pressure–volume relationship, is consistent with clinical measurements. An important aspect of this characterization is that it required no additional calibration and used only passive material parameters determined by Gültekin et al. (36) via triaxial shear tests of human left ventricular tissue specimens, which is an experimental approach that has been shown to be particularly effective in generating appropriate myocardial constitutive parameters (94), along with the chamber geometry and material axes. Our model also captures the Frank–Starling mechanism, as discussed further below. This confirms our model's ability to predict the complex effect of increased preload on stroke volume and tests both the passive and active material characterization in our framework. The Klotz curve and Frank–Starling results indicate that the ventricular material parameters, including the fiber fields, used in this work generate a mechanical response that is consistent with in vivo measurements. In addition, prior work has shown that in cardiac mechanics models with rule-based descriptions of the fiber architecture, it is important to use constitutive models for the atria and ventricle that account for fiber dispersion, as we do herein, to adequately capture ventricular function (93). In summary, the material parameters used in this study enable the model to generate physiologic dynamics as assessed through both qualitative analyses of model outputs as well as quantitative metrics.

### The Frank–Starling response

Simulations of beat-to-beat dynamics under changes in loading conditions demonstrate some of the predictive capabilities of the model. Most models of the heart that recapitulate the Frank–Starling mechanism use detailed electrophysiological models and incorporate tension-dependent loading into their models of active contraction (11, 14, 28, 96). We are unaware of other comprehensive models of cardiac fluid dynamics or FSI that have demonstrated this behavior while replicating multicycle in vivo experimental designs. One of the few prior simulation studies examining the Frank–Starling response is the work of Augustin et al. (97), which uses a comprehensive model of cardiac electromechanics but does not include a detailed model of cardiac fluid dynamics or FSI. In our model, the Frank–Starling response naturally arises following changes to preload, and it is not explicitly built into the contraction model. It is unknown whether this is inherent to the active strain approach to modeling muscle contraction used in our study or if it is also present in other contraction schemes, such as active stress (98). The ability to provide physiological responses to changes in loading is a critical feature of predictive models of cardiac dynamics. FSI approaches are needed to generate such responses in models of cardiac fluid dynamics.

Figure 9a shows that our model captures the physiological trend that larger end-diastolic volumes, or ventricular preload, result in larger stroke volumes. This trend is quantified in the ventricular function curve shown in Figure 9c. Figure 9b highlights that the end-diastolic filling section of the pressure–volume relation is very consistent across loads, which is also observed in vivo (20, 21). This section of the pressure–volume relation is often used as a surrogate for the static end-diastolic pressure–volume relation because it is difficult to obtain static end-diastolic pressure–volume curves in patients or human subjects (20, 21). An interesting finding of the present study is that the dynamic end-diastolic pressure–volume relationship inferred from the pressure–volume loops (Figure 9b) differs from the static end-diastolic pressure–volume curve generated by the model (Figure S2). This is not a shortcoming of the model, however. Instead, these differences are in agreement with Frank’s observations from frog experiments he performed in 1901 (99) as well results from more recent studies (100). We speculate that this difference is due to the momentum of ventricular wall after relaxation. This view is shared by other researchers who note that ventricular relaxation time has an effect on diastolic filling, which would impact this portion of the pressure–volume relation (99, 100). Supplementary Discussion Section *Analysis of the Frank-Starling response* provides additional commentary on the Frank–Starling response generated by the model heart.

### Model credibility

As of 2023 November, the U.S. Food and Drug Administration (FDA) finalized their recommendations for including first principles based models in medical device submissions (101), including those for in silico clinical trials, based on the V&V 40 guidelines introduced by The American Society for Mechanical Engineers in 2018 (102). The FDA recommendations outline methods for assessing the credibility of computational models, including their verification and validation, and per this guidance, emergent model behavior, or “evidence that demonstrates that the finalized computational model reproduces phenomena that are known to occur in the system at the specified conditions but were not pre-specified or explicitly modeled by the governing equations,” (101) is one of the eight key categories of credibility evidence suggested for computational models. The present study demonstrates the potential of our model to produce physiologic left heart dynamics with fully three-dimensional descriptions of the valves given physiologic operating conditions. The emergent model behavior described throughout this Discussion, such as the qualitative features of the atrial and ventricular pressure–volume loops, the valve dynamics and associated flow waveforms, metrics such as VFT, and the Frank–Starling response, support the credibility of this model with respect to appropriate physiologic behavior.

An important remark is that a model that reproduces physiological valvular flow rates or chamber volume dynamics alone is not guaranteed to correspond to a healthy heart. Indeed, patients with heart failure with preserved ejection fraction present with a normal ejection fraction, including appropriate end-systolic and end-diastolic volumes (103–105). This heart failure modality is commonly encountered in clinical practice, accounting for 40–50% of all heart failure cases with a prevalence of 1–5% in the general population (103, 104). Further, left ventricular hypertension develops as the disease progresses (105), but it is not necessarily accompanied by systemic hypertension (104). To assess if the function of our model is consistent with that of a healthy heart, we computed the model VFT (60) and the E-wave deceleration time (91), which are used as indicators of diastolic heart failure in vivo. Critically, these metrics were not calibrated but instead emerged from the complex interplay between the components of the model, and their values fall within healthy ranges, supporting the notion that our framework is capturing normal cardiac function.

Beyond emergent model behavior, earlier work (54, 55) has verified that the IFED method and its implementation generate appropriate benchmark mechanical responses for hyperelastic material models. However, it is clear that further verification and validation work is necessary to justify the application of this model contexts of use such as in silico clinical trials (101). Rigorous approaches to a verification and validation study of a model of this scale should proceed in a hierarchical manner and include isolated convergence studies for each of the valves as well as for the heart model as whole. Examples of these types of studies have also been conducted using the IFED method for porcine and bovine replacement aortic valves (57, 58), but that work serves more as a guideline for future validation work than a substitute, because the valve geometry, the finite-element mesh resolution, and the dynamic fluid viscosity are different from those used in the present work. Numerical convergence results for the specific aortic and mitral valve models used in our simulations are included in Supplementary Results Section *Grid convergence studies*. Further considerations must be made for the specific context of use in question and, critically, must reflect the needs of the study, such as resolving high fidelity resolution flow dynamics or less comprehensive studies that are primarily concerned with changes in chamber pressures and volumes. For example, whereas the streamlines reproduced in Figure 8 capture the qualitative features from in vivo velocity studies and provide evidence of model credibility, it would be inappropriate to suggest the velocity fields produced by the model are thereby fully validated. Hence, a next step in establishing the utility of this model in a clinical setting would be a comprehensive verification and validation study using a new model of cardiac anatomy and physiology corresponding to a subject for which additional in vivo flow measurements, such as 4D flow MRI, are available. Such a study would be a natural extension but is beyond the scope of the present work.

## Conclusions

Overall, because the dynamics of our heart model all result from mechanistic interactions among the structures of the heart and the blood, the level of agreement with existing clinical and experimental data is excellent. Indeed, a strength of the model is that it captures the subtleties of in vivo pressure–flow relations, such as the closing transients and resultant volume changes, rather than emulating ideal pressure–volume curves that are achieved by noncompliant diode-like descriptions of the valves. Further, a major advantage of our model is its ability to permit direct and simultaneous access to metrics such as flows through the valve annuli and localized pressures. Such data are critical for clinical applications, e.g. simulation studies of treatments for structural heart disorders such as mitral valve regurgitation. The discussion of translational applications for this model is centered on valvular disorders because, unlike previous whole heart models, this model has fully three-dimensional descriptions of the valves with material properties that reflect human valvular tissue. Indeed, valvular heart diseases represent a substantial and rapidly growing burden on public health throughout the world (106). These include rheumatic heart disease, which primarily affects young people between the ages of 5 and 15 and causes permanent damage to one or more heart valves, along with aortic stenosis and mitral regurgitation, which are mainly diseases of the elderly. This model is also relevant for mechanistic studies of cardiac dysfunction, such as reduced ventricular compliance from cardiomyopathy and discordant or nonextant contraction following acute myocardial infarction, and congenital heart defects, such as those within the spectrum of single ventricle physiology. In these cases, our framework could lead to insights into experimental treatments and surgeries that can be used to improve clinical care and corresponding outcomes.

## Supplementary Material

pgae392_Supplementary_Data

## Data Availability

All data supporting the conclusions of this study are included in the manuscript and supporting information. The model was built using the IBAMR software infrastructure available on GitHub (ibamr.github.io), which is released under a permissive open-source license.

## Preprints

This manuscript was first published as a preprint, Davey M, et al. (2023) Simulating Cardiac Fluid Dynamics in the Human Heart. *arXiv* 2307.02680, available from https://doi.org/10.48550/arXiv.2307.02680.
