# Supplementary material for: Simulating cardiac fluid dynamics in the human heart
Source: PNAS Nexus. 2024 Sep 10;3(10):pgae392. doi: 10.1093/pnasnexus/pgae392 (PMC11492567; doi:10.1093/pnasnexus/pgae392)
Supplement: pgae392_Supplementary_Data [file pgae392_supplementary_data.zip › supplementary_information.pdf]

SUPPLEMENTARY INFORMATION

Methods

Anatomical model

The anatomy of the heart chambers and the nearby great vessels were reconstructed from cardiac CT images (voxel size 0.32 mm×0.32 mm×0.4 mm) of a healthy adult male provided by Siemens Healthineers. All data were fully deidentified by Siemens, and the study team has no way to determine the identity of the subject. Chamber reconstruction used methods previously detailed by Segars et al.[1,2] Because the cardiac valves were obscured in the CT images, we generated idealized anatomical models of the valves with dimensions that are consistent with prior experimental studies of human heart valves. The aortic valve leaflet geometry[3] reflects the sinus height,[4] valve diameter,[5] lunulae coaptation height,[5] and leaflet thickness[6] of human aortic valves. The pulmonary valve leaflets are a replica of the aortic valve leaflets that were scaled to fit within the model pulmonary artery. The mitral valve leaflet surface geometry was built using a set of parametric equations derived from mitral valve imaging data,[7] which was further modified to match the thickness[8] and length[9] of human mitral valves. The tricuspid valve leaflet geometry was based on valve dissection studies[10,11] and has three identical leaflets that were adjusted for length to ensure closure during systole. The papillary muscles were placed in locations identified in the CT images and connected to the atrioventricular valves by chordae tendineae. Marginal chordae were uniformly distributed along the mitral and tricuspid valve leaflet edges, and strut and basal chordae were added to the mitral valve leaflets to prevent prolapse.[12] These valve models were subsequently merged with the CT-derived chamber anatomy, yielding the model illustrated in Figure 1(a).

We used TetWild[13] to construct a monolithic, conforming tetrahedral mesh that includes the myocardium, valves, cardiac skeleton, and great vessels. The mesh used by our model contains approximately 2.4M elements with an average diameter of 1.17 mm. The mesh is partitioned into subdomains, which are groups of elements that share the same constitutive model and material properties. Subdomains defined in the model include: the left and right atria; the left and right ventricles; the aortic and pulmonary valves; the mitral and tricuspid valves, including the valve cusps, chordae tendenae, and papillary muscles; the cardiac skeleton; and short segments of the great vessels, including the ascending aorta, pulmonary artery, superior and inferior vena cavae, and pulmonary veins. Because these structures are all described within a single conforming mesh, no additional mechanical coupling conditions need to be imposed along interfaces between subdomains. The anatomy captured in the model fits within a bounding box with dimensions 20 cm×11.6 cm×17.4 cm, with the longest dimension corresponding to the distance between the branch termini of the pulmonary artery. Figure S1 shows two perspectives of the heart mesh and selected subdomains, with a focus on the left ventricle and mitral valve apparatus.

Cardiac biomechanics models

Fiber architecture

To describe the local material coordinate directions that determine the tissue anisotropy, we created an orthonormal reference frame $\{\mathbf{e}_\mathrm{f}, \mathbf{e}_\mathrm{s}, \mathbf{e}_\mathrm{n}\}$ in each element of the structural mesh. Briefly, in the myocardium, $\mathbf{e}_\mathrm{f}$ is the principal myofiber orientation, $\mathbf{e}_\mathrm{s}$ points in the transmural direction (from the endocardium to the epicardium), and $\mathbf{e}_\mathrm{n} = \mathbf{e}_\mathrm{f} \times \mathbf{e}_\mathrm{s}$. The material axes in the valve leaflets capture the collagen fiber architecture identified in human heart valves.[14,15] The $\mathbf{e}_\mathrm{f}$ direction field describes the mean collagen fiber orientation and runs from commissure to commissure in each valve, and $\mathbf{e}_\mathrm{s}$ runs radially from the valve ring, where the leaflet intersects the myocardium, to the free edge. To account for fiber angle dispersion within the valve leaflets, our valve biomechanics models use two distinguished collagen fiber directions, $\mathbf{e}_{\mathrm{f}^\pm} = \cos(\theta_\mathrm{f})\mathbf{e}_\mathrm{f} \pm \sin(\theta_\mathrm{f})\mathbf{e}_\mathrm{s}$. These directions are rotations of $\mathbf{e}_\mathrm{f}$ about $\mathbf{e}_\mathrm{n} = \mathbf{e}_\mathrm{f} \times \mathbf{e}_\mathrm{s}$ by the angle $\pm\theta_\mathrm{f}$, which is a material parameter fit to experimental data. In the chordae tendineae, $\mathbf{e}_\mathrm{f}$ is aligned with the long axis of each chord. Figure 1(c–d) visualize the myocardial fiber directions, and Figure 1(e) shows the valve leaflets' mean collagen fiber direction with the fibers for the chordae and papillary muscles.

We use a harmonic interpolation technique that has been widely adopted in model-based approaches to describe cardiac fiber architecture.[16] Specifically, on each subdomain of the structure's reference configuration Ω_0^s, we construct functions $\phi(\mathbf{X})$ that satisfy $\nabla^2\phi(\mathbf{X}) = 0$, with $\mathbf{X} \in \Omega_0^\mathrm{s}$, and we use each resulting function to determine a local coordinate direction via $\nabla\phi(\mathbf{X})/\|\nabla\phi(\mathbf{X})\|$. The orientation of each direction field is controlled through boundary conditions for $\phi(\mathbf{X})$ that are imposed along the subdomain boundaries. For instance, to model a group of fibers that originates on one part of the subdomain boundary and terminates along another part of the boundary, we respectively impose the Dirichlet boundary conditions $\phi(\mathbf{X}) = 0$ along the origin and $\phi(\mathbf{X}) = 1$ along the terminus. To prevent fibers from passing through a part of the subdomain boundary, we impose homogeneous Neumann boundary conditions, $\partial\phi(\mathbf{X})/\partial\mathbf{N} = 0$, in which $\mathbf{N} = \mathbf{N}(\mathbf{X})$ is the unit normal to the subdomain boundary.

The ventricular material axes follow the rule-based method of Rossi et al.,[17] which reflects experimentally characterized relationships between fiber angle and transmural position within the ventricular myocardium.[18–20] In this approach, the sheet axis $\mathbf{e}_\mathrm{s}$ is generated first by the harmonic interpolation procedure with boundary conditions $\phi(\mathbf{X}) = 0$ on the endocardium and $\phi(\mathbf{X}) = 1$ on the epicardium, which produces a transmurally oriented direction field. A reference direction field that runs from the heart's apex to the mitral and tricuspid annuli through the ventricular myocardium is generated in each ventricle as $\mathbf{e}_{\mathrm{n}_0} = \mathbf{c} - (\mathbf{c} \cdot \mathbf{s}_0)$, in which $\mathbf{c}$ is the vector pointing from the apex to the center of the chamber's atrioventricular valve ring. An initial circumferential field is then created by setting $\mathbf{e}_{\mathrm{f}_0} = \mathbf{e}_\mathrm{s} \times \mathbf{e}_{\mathrm{n}_0}$. Next, $\mathbf{e}_{\mathrm{f}_0}$ is rotated about the $\mathbf{e}_\mathrm{s}$ axis to capture transmural fiber rotation according to rules[17,21] based on histology studies of the ventricular myocardium[18–20] to generate the myofiber orientation $\mathbf{e}_\mathrm{f}$. Finally, we set $\mathbf{e}_\mathrm{n} = \mathbf{e}_\mathrm{f} \times \mathbf{e}_\mathrm{s}$.

The myofiber architecture of the atria is substantially more complex than the ventricular fiber structure, but prior studies have identified subregions within the atria with distinct principal myofiber orientations that are amenable to rule-based fiber models.[22–24] Herein we use a version of the rule-based approach detailed by Rossi et al.[25] As with the ventricles, a transmural direction field $\mathbf{e}_\mathrm{s}$ is generated by setting the boundary conditions $\phi(\mathbf{X}) = 0$ on the endocardial surface and $\phi(\mathbf{X}) = 1$ on the epicardial surface. The atria are then partitioned into anatomical subregions. Within each

subregion, the principal myofiber orientation is known, and subdomain boundary conditions are applied to generate the required myofiber field $\mathbf{e}_\mathrm{f}$. Finally, we set $\mathbf{e}_\mathrm{n} = \mathbf{e}_\mathrm{f} \times \mathbf{e}_\mathrm{s}$, as in the ventricles.

The method for generating the collagen fiber network in the valves is adopted from Hasan et al.[3] The transmural direction field $\mathbf{e}_\mathrm{n}$ is generated by setting $\phi(\mathbf{X}) = 0$ on the leaflet surface facing the ventricle and $\phi(\mathbf{X}) = 1$ on the leaflet surface facing the great vessels and the atria for the semilunar valves and atrioventricular valves, respectively. A radial direction field, $\mathbf{e}_\mathrm{s}$, is generated in each leaflet by setting $\phi(\mathbf{X}) = 0$ on the edge where the leaflet intersects with the myocardium and $\phi(\mathbf{X}) = 1$ along the leaflet's free edge. The circumferential direction field corresponding to the mean collagen fiber axis is then determined as $\mathbf{e}_\mathrm{f} = \mathbf{e}_\mathrm{s} \times \mathbf{e}_\mathrm{n}$. The chordae tendineae fiber axes $\mathbf{e}_\mathrm{f}$ are captured by setting $\phi(\mathbf{X}) = 0$ on the surfaces where the chordae meet the papillary muscles and $\phi(\mathbf{X}) = 1$ on the surfaces where the chordae meet the valve leaflets.

Material characterization

The biomechanical responses of all major structures of the heart are described using the framework of large deformation elasticity. Briefly, Ω_0^s is a Lagrangian reference coordinate system attached to the initial configuration of the heart, and Ω_t^s is the current configuration at time t. The deformation mapping $\chi : (\Omega_0^\mathrm{s}, t) \mapsto \Omega_t^\mathrm{s}$ relates reference and current coordinates, so that $\chi(\mathbf{X}, t) \in \Omega_t^\mathrm{s}$ is the current position of $\mathbf{X} \in \Omega_0^\mathrm{s}$ at time t. The mechanical responses of all structural components are defined by hyperelastic energy functionals $\mathcal{W}$ of the deformation gradient tensor $\mathbb{F}(\mathbf{X}, t) = \partial \chi(\mathbf{X}, t)/\partial \mathbf{X}$.[21] We describe myocardial contractile mechanics using an active strain approach,[26] which assumes that the Helmoltz free energy $\mathcal{W}$ can be expressed using $\mathbb{F}$ and an internal variable $\mathbb{F}_\mathrm{A}$ that represents the active component of the deformation, yielding $\mathcal{W} = \mathcal{W}(\mathbb{F}, \mathbb{F}_\mathrm{A})$. The active strain model links $\mathbb{F}$ and $\mathbb{F}_\mathrm{A}$ through an intermediate virtual configuration, so that $\mathbb{F} = \mathbb{F}_\mathrm{E}\mathbb{F}_\mathrm{A}$, and it assumes that the energy can be defined in the intermediate configuration, such that $\mathcal{W}(\mathbb{F}, \mathbb{F}_\mathrm{A}) = \mathcal{W}(\mathbb{F}_\mathrm{E}) = \mathcal{W}(\mathbb{F}\mathbb{F}_\mathrm{A}^{-1})$.[17] Additional discussion of active strain models of contraction is provided in Materials Section *Active contraction*.

Different strain-energy functionals are used for different structures to reflect their specific material properties. Following the principle of material objectivity, the hyperelastic models are formulated using the right Cauchy-Green strain tensor, $\mathbb{C} = \mathbb{F}^\mathsf{T}\mathbb{F}$, in terms of $I_1 = \mathrm{tr}(\mathbb{C})$, $I_{4i} = \mathbf{e}_i^\mathsf{T}\mathbb{C}\mathbf{e}_i$, $I_{4i}^\star = \max(I_{4i}, 1)$, and $I_{8ij} = \mathbf{e}_i^\mathsf{T}\mathbb{C}\mathbf{e}_j$, in which i and j index the material coordinate axes.

The ventricles and papillary muscles use the orthotropic Holzapfel-Ogden model,[21]

$$\mathcal{W} = \frac{a}{2b}\exp(b(I_1-3)) + \sum_{i=\mathrm{f,s}} \frac{a_i}{2b_i}(\exp(b_i(\kappa_i I_1 + (1-3\kappa_i)I_{4i}-1)^2)-1) + \frac{a_\mathrm{fs}}{2b_\mathrm{fs}}(\exp(b_\mathrm{fs} I_{8\mathrm{fs}}^2)-1). \quad \text{(S1)}$$

We use material parameters from Gültekin et al.[27] that are based on triaxial shear tests on cuboid specimens of human left ventricles. The atria use the model of Augustin et al.,[28]

$$\mathcal{W} = \frac{a}{2b}(\exp(b(I_1-3)) - 1) + \frac{a_\mathrm{f}}{2b_\mathrm{f}}(\exp(b_\mathrm{f}(\kappa I_1 + (1-3\kappa)I_{4\mathrm{f}} - 1)^2) - 1). \quad \text{(S2)}$$

We use material parameters from Augustin et al.[28] that were calibrated using biaxial strain test data from anterior and posterior specimens of human left atria. The parameters κ_i in Eq. (S1) and κ in Eq. (S2) characterize myofiber angle dispersion.[21,28]

The valve leaflets use a version of the Holzapfel-Gasser-Ogden model[29] by Murdock et al.,[30]

$$\mathcal{W} = a\{\exp[b(I_1 - 3)] - 1\} + \frac{a_\mathrm{f}}{2b_\mathrm{f}} \sum_{k\in\{+,-\}} \{\exp[b_\mathrm{f}(I^\star_{4\mathrm{f}^k} - 1)^2] - 1\}. \tag{S3}$$

We fit the parameters for all valves using biaxial stress-strain data generated by Pham et al.,[31] as described below. Notice that the collagen fiber stresses in our leaflet models engage in tension but not in compression, which corresponds to the concept that collagen fibers collapse under compression and do not substantially contribute to the stress response of the material.[32] The chordae tendineae use a nonlinear spring model,

$$\mathcal{W} = \frac{a}{2}(I_1 - 3) + \frac{a_\mathrm{f}}{3}\left(I^\star_{4\mathrm{f}} - 1\right)^3. \tag{S4}$$

We determined mitral chordae parameters for the posterior and anterior leaflet chordae from uniaxial stress-strain tests of human mitral chordae,[33] as described below. We also determined tricuspid chordae parameters from uniaxial stress-strain tests of human tricuspid chordae;[34] all tricuspid chordae use the same parameters because of limited availability of human tissue data. Briefly, to determine material parameters for the chordae and the valves, we extracted stress-strain curves using WebPlotDigitizer[35] and fit the constitutive model using `lsqcurvefit` in MATLAB (MathWorks, Natick, MA) with a function tolerance of 1e-12 and the lower bounds for all parameters set at zero to prevent negative values. Figure S4 shows our model fits.

To avoid severe mitral valve regurgitation, we prestrained some chordae along their major fiber axes. This was necessary because the model construction process produced some loose chordae, which led to valve prolapse during ventricular systole. We used a prestraining approach that is equivalent to the active strain formulation for the myocardium,[26] but with fiber stretch γ_f constant in time. The fiber stretch terms for the eighteen mitral valve chordae range in values from 0.0 to 0.25, with the majority set to 0.15.

The vena cavae, pulmonary veins, ascending aorta, and pulmonary artery are modeled as neo-Hookean materials,

$$\mathcal{W} = \frac{a}{2}(I_1 - 3). \tag{S5}$$

Model parameters were chosen to allow for realistic vessel compliance while avoiding excessive deformation across the cardiac cycle.

Table S1 lists all constitutive model parameters.

Active contraction

As stated above, we use an active strain formulation to describe the active deformations of the cardiac muscle. In this model, the deformation gradient is decomposed such that $\mathbb{F} = \mathbb{F}_\mathrm{E}\mathbb{F}_\mathrm{A}$. This decomposition can be interpreted as follows: The muscle tissue is subdivided in microscopic elements, and each of these elements undergoes a change in configuration described by the active deformation gradient tensor $\mathbb{F}_\mathrm{A}$. The configuration described by the active deformation gradient is a virtual, potentially non-compatible intermediate configuration. For the muscle to achieve its contracted configuration, a subsequent elastic deformation $\mathbb{F}_\mathrm{E}$ is required. The elastic deformation gradient describes the deformations necessary to put the contracted elements back together while also satisfying the balance of momentum with the corresponding boundary conditions. We give a

**Tissue**	a **[kPa]**	b	a_f **[kPa]**	b_f	κ_f	θ_f **[rad]**	a_s **[kPa]**	b_s	κ_s	a_{fs} **[kPa]**	b_{fs}
Ventricles[27]	0.4	6.55	3.05	29.05	0.08	–	1.25	36.65	0.09	0.15	6.28
Papillary Muscles[27]	0.4	6.55	3.05	29.05	0.08	–	1.25	36.65	0.09	0.15	6.28
Atria[28]	2.92	5.6	11.84	17.95	0.17	–	–	–	–	–	–
Aortic Valve[31]	0.172	25.24	10.2	384.6	–	0.1055	–	–	–	–	–
Anterior Mitral Leaflet[31]	0.398	14.91	11.1	104.9	–	0.7746	–	–	–	–	–
Posterior Mitral Leaflet[31]	0.24	15.63	2.462	84.1	–	0.0	–	–	–	–	–
Pulmonary Valve[31]	0.2135	11.14	0.9634	49.66	–	0.0001	–	–	–	–	–
Tricuspid Valve[31]	0.1502	15.23	0.3157	40.73	–	0.0058	–	–	–	–	–
Anterior Mitral Basal Chordae[33]	5.718e3	–	2.26e5	–	–	–	–	–	–	–	–
Anterior Mitral Marginal Chordae[33]	1.619e4	–	1.679e5	–	–	–	–	–	–	–	–
Anterior Mitral Strut Chordae[33]	1.0e3	–	2.652e5	–	–	–	–	–	–	–	–
Posterior Mitral Basal Chordae[33]	4.796e3	–	1.904e5	–	–	–	–	–	–	–	–
Posterior Mitral Marginal Chordae[33]	1.105e4	–	3.177e5	–	–	–	–	–	–	–	–
Tricuspid Valve Chordae[34]	4.091e4	–	1.66e5	–	–	–	–	–	–	–	–
Aorta	1.2e2	–	–	–	–	–	–	–	–	–	–
Vena Cavae	1.43e2	–	–	–	–	–	–	–	–	–	–
Pulmonary Artery	1.0e2	–	–	–	–	–	–	–	–	–	–
Pulmonary Veins	1.43e2	–	–	–	–	–	–	–	–	–	–

Table S1: Citations are included for the origin of the data that were used for the parameter fitting or the parameters themselves. As stated in the main text, the material parameters for the great vessels are used to prevent gross deformations and are not based on human tissue studies. The numerical bulk modulus β_s is set to 4.0e4 kPa throughout the structure.

Chamber	t_{delay} (s)	t_{peak} (s)	t_{plateau} (s)	t_{drop} (s)	$\gamma_{\text{f,max}}$
Atria	0.0	0.25	0.01	0.3	0.08
Ventricles	0.18[43]	0.45	0.01	0.3	0.3

Table S2: Parameters for the active strain model. The contraction period is 1 second.

short description of the model below, but refer the interested reader to the large literature on the active strain formulation.[17,26,36–42] We also note that there is an abundance of different contraction formulations in the literature, and at present, there is no consensus model.

In the active strain formulation, $\mathbb{F}_\text{A}$ defines the time-dependent change in the reference configuration resulting from muscle contraction.[26] We use

$$\mathbb{F}_\text{A} = \mathbb{I} + \gamma_\text{f}\, \mathbf{e}_\text{f} \otimes \mathbf{e}_\text{f} + \gamma_\text{s}\, \mathbf{e}_\text{s} \otimes \mathbf{e}_\text{s} + \gamma_\text{n}\, \mathbf{e}_\text{n} \otimes \mathbf{e}_\text{n}, \tag{S6}$$

in which γ_i defines the deformation scaling along each material axis $i \in \{\text{f,s,n}\}$. The contraction of the myocardium is assumed to be volume-preserving, so $\det(\mathbb{F}_\text{A}) = 1$, and, for simplicity, transversely isotropic along the fiber axis $\mathbf{e}_\text{f}$. We prescribe $\gamma_\text{f}(t)$ and thereby obtain $\gamma_\text{n}(t) = \gamma_\text{s}(t) = (1 + \gamma_\text{f}(t))^{-1/2} - 1$.

The contraction timings for the atria and ventricles are based on studies of conduction propagation through the atria, atrioventricular node, and ventricles,[43] as well as pressure profiles measured within the chambers.[44] We defined the activation waveform by the function

$$g(t) = \begin{cases} \frac{1}{2} - \frac{1}{2}\cos\left(\frac{\pi}{t_\text{peak}} t\right) & \text{if } t - t_\text{delay} < t_\text{delay}, \\ 1 & \text{if } \text{t}_\text{peak} \leq t - t_\text{delay} \leq t_\text{plateau} + t_\text{peak}, \\ \frac{1}{2} + \frac{1}{2}\cos\left(\frac{\pi}{t_\text{drop}}(t - t_\text{peak} - t_\text{plateau})\right) & \text{if } t_\text{plateau} + t_\text{peak} < t - t_\text{delay} < t_\text{plateau} + t_\text{peak} + t_\text{drop}, \end{cases} \tag{S7}$$

in which t_delay is the time from the beginning of the cycle to the start of contraction, t_peak is the time to peak contraction after the onset of contraction, t_plateau is the time spent at peak contraction, and t_drop is the time from the end of peak contraction to no contraction. Together with the magnitude of peak contraction, $\gamma_\text{f,max}$, we have $\gamma_\text{f}(t) = \gamma_\text{f,max}\, g(t)$. The active strain parameters for the atria and the ventricles are stated in Table S2, and the respective waveforms are visualized in the bottom right panel of Figure 2. The majority of the parameters were acquired through manual calibration except for the ventricular t_delay, which was chosen to correspond to the time it takes for the activation signal to propagate from the sinoatrial node to the ventricles.[43]

Pericardium

Following the approach of Pfaller et al.,[45] the force from the pericardium, $\mathbf{F}(\mathbf{X}, t)$, imposed on the epicardial surface, is determined by a system of distributed damped springs via

$$\mathbf{F}(\mathbf{X}, t) = \mathbf{n}(\mathbf{X}, t) \otimes \mathbf{n}(\mathbf{X}, t)\, [\kappa\, (\mathbf{X} - \chi(\mathbf{X}, t)) - \eta\, \mathbf{U}(\mathbf{X}, t)]\,, \tag{S8}$$

in which $\mathbf{n}(\mathbf{X}, t)$ is the surface unit normal to the epicardium in the current configuration, $\mathbf{U}(\mathbf{X}, t)$ is the velocity in the current configuration of material point $\mathbf{X}$, κ is a tethering constant, and η

is a damping constant. Figure 1(b) shows a schematic of the pericardium model superimposed with the full heart geometry. In our simulations, the parameters for the pericardium model are $\kappa = 1.0$ kPa/mm and $\eta = 5.0$e-2 kPa·s/mm. These parameters were chosen to limit gross epicardial oscillations, especially during ventricular relaxation. As the pericardial tethering model only acts normal to the epicardial surface, it is a weak constraint on the chambers that freely permits tangential motion.

Blood and circulation

At the length scale of the heart, blood behaves like a Newtonian fluid,[46] and the dynamics of blood are well approximated by the incompressible Navier-Stokes equations. We choose uniform mass density $\rho = 1.0\ \mathrm{g \cdot cm^{-3}}$ and uniform dynamic viscosity $\mu = 4\ \mathrm{mPa \cdot s}$.[47]

The afterload provided by the systemic and pulmonary circulations are described using three-element Windkessel models applied at locations where the ascending aorta (Ao) and the left and right pulmonary artery branches (LPA and RPA) intersect the boundary of the computational domain.[48,49] The state variable for each of these models is the afterload pressure, downstream of the great vessel, that satisfies the equation

$$C_i \frac{\mathrm{d}P_{\mathrm{wk},i}}{\mathrm{d}t} + R_{\mathrm{p},i}\, P_{\mathrm{wk},i} = Q_i, \quad i \in \{\mathrm{Ao, LPA, RPA}\}. \tag{S9}$$

Q_i is the volume of blood per unit time flowing between vessel i and the corresponding Windkessel model at the boundary of the computational domain, with positive values corresponding to outflow and negative values corresponding to inflow. Model calibration was performed by querying the outflow rate waveforms and adjusting the peripheral resistance and compliance terms to maintain physiologic values of the systolic and diastolic pressures at the boundary[44,50,51] that mimic the systemic pressure downstream of the ascending aorta following a procedure described below. The models of the peripheral circulations used in this study are not closed, and flows and pressures from the left and right sides of the heart are uncoupled. Instead, venous return is modeled by a pressure-driven flow source located in each atrium (LA and RA).[52] The flow sources are determined by

$$L_j \frac{\mathrm{d}Q_j}{\mathrm{d}t} + R_j\, Q_j = P_{\mathrm{source},j} - P_j, \quad j \in \{\mathrm{LA, RA}\}, \tag{S10}$$

in which $P_{\mathrm{source},j}$ is the pressure upstream in the vena cava ($\mathrm{P_{VC}}$) for the right atrium and the pressure upstream in the pulmonary veins ($\mathrm{P_{PV}}$) for the left atrium (see Figure 1), which are treated as constant, and P_j is the pressure sampled within the chamber. The ratio of the parameters L_j and R_j governs the timescale of the flow response to the difference between the source and atrium pressures. Figure 1(b) shows the Windkessel circulation models and the flow sources in relation to the full heart geometry.

Parameters for the aorta and the pulmonary arteries are provided in Table S3. The characteristic resistance value for the aorta was taken from Sturgiopoulos et al.[49] The peripheral resistance and compliance were calibrated to fit specific flows generated from contraction of the left ventricle. The first step of this calibration procedure was to calculate the flow rate waveform at the intersection of the aorta with the edge of the computational domain; see Figure S5(a). The parameters were then adjusted to generate physiologic systolic and diastolic pressure values,[44] or the maximum and

Vessel	R_C (mmHg·s/mL)	R_P (mmHg·s/mL)	C (mL/mmHg)
Aorta	0.033[49]	1.71	1.1
Pulmonary Arteries	0.0219	0.08	5.56

Table S3: The systemic circulation Windkessel parameters are tuned to the flow rate output at the edge of the ascending aorta. The pulmonary circulation Windkessel parameters are based on in vivo pressure measurements.

Chamber	L (mmHg·s^2/mL)	R (mmHg·s/mL)	P_{source} (mmHg)
Right Atrium	300·Δt	0.15	3.75
Left Atrium	240·Δt	0.15	10

Table S4: Parameters for the flow sources in the left and right atria.

minimum pressure values, respectively. Figure S5(b) shows the Windkessel model predictions of the pressure waveform in the aorta as well as the pressure waveform downstream from the characteristic resistance. The model fit was tested, as illustrated in Figure S5(c), by comparing the predicted pressure from the Windkessel model and the observed pressure at the intersection of the aorta and the computational domain. Over successive cycles, the pressure range generated by the model converges to the pressure wave predicted by the specified Windkessel model parameters. This procedure mimics the baroreceptor reflex, which is a physiological control mechanism that adjusts vascular tone to maintain physiological blood pressure.[50]

Parameters for the pulmonary arteries were based on pulmonary circulation values. To calculate nominal values for these parameters, we assumed a cardiac output of 100 mL/s. With the heart rate defined to be 60 BPM, the stroke volume is 100 mL. The pulmonary arteries were assumed to have a systolic pressure of 19 mmHg and diastolic pressure of 10 mmHg, with a mean pulmonary arterial pressure of 13 mmHg and a mean pulmonary venous pressure of 9 mmHg.[51] We assumed the mean pressure drop from the pulmonary arteries to the pulmonary veins is completely described by the peripheral resistance, and that the flow rate was equally split between the two pulmonary arteries. This resulted in a nominal value for the peripheral resistance of R_p = 0.08 mmHg·s/mL. The compliance value was calculated as the fraction of the stroke volume entering the artery divided by the pulse pressure. This resulted in a compliance value of 5.56 mL/mmHg. The pulmonary characteristic resistance was chosen to be of comparable magnitude to but smaller than the aortic characteristic resistance.

The flow source parameters were also determined empirically. The inertance of each source, which we have found to be most important with regards to maintaining numerical stability, is chosen to be time-step size dependent, and it is set to be as small as possible while preventing spurious changes in flow rate.[52] Table S4 lists the values for the source parameters used in the present study.

Fluid-structure interaction

Our model uses an immersed approach to simulating fluid-structure interaction (FSI). The immersed boundary (IB) method,[53] originally introduced by Peskin to model the fluid dynamics of heart

valves,[54,55] is the earliest example of such a numerical method. It treats fluid-structure systems in which an elastic structure is immersed in a viscous incompressible fluid. The IB method describes the structure in Lagrangian form and the fluid in Eulerian form, and it uses integral equations with Dirac delta function kernels to connect the Lagrangian and Eulerian frames. When the governing equations are discretized for computer simulation, the singular delta function is replaced by a regularized version of the delta function. Our computations use an efficient nodal version[56] of a stabilized immersed finite element/difference (IFED) method.[56–59] This scheme is a variation on the IB method that uses a finite element description of the structure, enabling structural models with complex geometries and realistic constitutive models. The IFED method also employs a regularized version of the Dirac delta function, and the choice of delta function used in this study follows results from a recent benchmarking study.[58] The remainder of this section outlines the IFED method and provides details on numerical discretization parameters used to generate simulation results.

Briefly, the IFED method predicts the coupled dynamics of the fluid-structure system within a computational domain Ω that is partitioned into non-overlapping fluid and solid subdomains, Ω_t^{f} and Ω_t^{s}, that are indexed by time t. To enable the use of fast structured-grid solvers, we require that $\Omega = \Omega_t^{\mathrm{f}} \cup \Omega_t^{\mathrm{s}}$ is a fixed rectangular region. Our simulations use a computational domain of size 20 cm×20 cm×20 cm, which is slightly larger than the bounding box that contains the reconstructed anatomy. The IFED formulation uses both Eulerian variables, which are described using fixed physical coordinates $\mathbf{x} \in \Omega$, and Lagrangian variables, which are described using material coordinates $\mathbf{X}$ that are chosen to be the initial coordinates of the structure at time $t = 0$, so that $\mathbf{X} \in \Omega_0^{\mathrm{s}}$. The deformation mapping $\chi : (\Omega_0^{\mathrm{s}}, t) \mapsto \Omega_t^{\mathrm{s}} \subseteq \Omega$ connects reference and current coordinates, so that $\chi(\mathbf{X}, t) \in \Omega_t^{\mathrm{s}}$ is the current position of material point $\mathbf{X}$ at time t.

The equations of motion for the coupled fluid-structure system are

$$\rho \frac{\mathrm{D}\mathbf{u}}{\mathrm{D}t}(\mathbf{x}, t) = -\nabla p(\mathbf{x}, t) + \mu \nabla^2 \mathbf{u}(\mathbf{x}, t) + \mathbf{f}(\mathbf{x}, t), \qquad \mathbf{x} \in \Omega, \tag{S11}$$

$$\nabla \cdot \mathbf{u}(\mathbf{x}, t) = q(\mathbf{x}, t), \qquad \mathbf{x} \in \Omega, \tag{S12}$$

$$\mathbf{f}(\mathbf{x}, t) = \int_{\Omega_0^{\mathrm{s}}} \mathbf{F}(\mathbf{X}, t)\, \delta(\mathbf{x} - \chi(\mathbf{X}, t))\, \mathrm{d}\mathbf{X}, \qquad \mathbf{x} \in \Omega, \tag{S13}$$

$$q(\mathbf{x}, t) = \sum_j Q_j(t)\, \Psi_w(\mathbf{x} - \mathbf{Y}_j(t)), \qquad \mathbf{x} \in \Omega, \tag{S14}$$

$$\frac{\partial \chi}{\partial t}(\mathbf{X}, t) = \mathbf{U}(\mathbf{X}, t) = \int_{\Omega} \mathbf{u}(\mathbf{x}, t)\, \delta(\mathbf{x} - \chi(\mathbf{X}, t))\, \mathrm{d}\mathbf{x}, \qquad \mathbf{X} \in \Omega_0^{\mathrm{s}}, \tag{S15}$$

$$P_j(t) = \int_{\Omega} p(\mathbf{x}, t)\, \Psi_w(\mathbf{x} - \mathbf{Y}_j(t))\, \mathrm{d}\mathbf{x}, \qquad j \in \{\mathrm{LA}, \mathrm{RA}\} \tag{S16}$$

in which $\mathbf{u}(\mathbf{x}, t)$ and $\mathbf{U}(\mathbf{X}, t)$ are Eulerian and Lagrangian velocity fields, $p(\mathbf{x}, t)$ is the pressure, $\mathbf{f}(\mathbf{x}, t)$ and $\mathbf{F}(\mathbf{X}, t)$ are Eulerian and Lagrangian elastic force densities, $q(\mathbf{x}, t)$ and $Q_j(t)$ are Eulerian and Lagrangian fluid source distributions associated with the venous flow sources, which are determined from the reduced-order model specified in Eq. (S10), $P_j(t)$ are the pressures within the venous flow sources, $\mathbf{Y}_j(t)$ are the time-dependent positions of the venous flow sources, which are determined by computing averages of the current positions at time t of selected atrial mesh nodes, ρ is the mass density of the fluid-structure system, μ is the viscosity, $\delta(\mathbf{x})$ is the Dirac delta function, and $\Psi_w(\mathbf{x})$ is a smooth distribution function with finite support that integrates to 1 and associated length scale w. The Lagrangian elastic force density is defined in terms of the first Piola-Kirchhoff

structural stress tensor, $\mathbb{P}(\mathbf{X},t) = \frac{\partial \mathcal{W}}{\partial \mathbb{F}}(\mathbf{X},t)$, by requiring $\mathbf{F}(\mathbf{X},t)$ to satisfy

$$\int_{\Omega_0^s} \mathbf{F}(\mathbf{x},t) \cdot \boldsymbol{V}(\mathbf{X})\,\mathrm{d}\mathbf{X} = -\int_{\Omega_0^s} \mathbb{P}(\mathbf{X},t) : \nabla_{\mathbf{X}} \boldsymbol{V}(\mathbf{X})\,\mathrm{d}\mathbf{X} \tag{S17}$$

for all smooth vector-valued test functions $\boldsymbol{V}(\mathbf{X})$. See Boffi et al.[60] and Griffith and Luo[57] for additional discussion.

The arteries and veins of the heart model are connected to descriptions of the systemic and pulmonary circulation, as discussed in Section *Modeling human cardiac anatomy and physiology*, using the specific reduced models detailed in Methods Section *Blood and circulation*. A simple time-step splitting scheme is used to advance the detailed and reduced model state variables, as described previously.[48,52] Notice that fluid pressures within the sources are determined by sampling $p(\mathbf{x},t)$ using the same function $\Psi_w(\mathbf{x})$ that is used to distribute the source strengths Q_j. For the arteries, we use a coupling scheme that relates pressures and flow rates at the vessel outlets, which are attached to portions of $\partial\Omega$, the boundary of the computational domain.[48,61] Briefly, we use a Strang-type fractional time stepping scheme, in which the state variables of the detailed and reduced-order models are advanced separately. The flow rate generated by the detailed model at the outlet is used as an input to the circulation model and drives its pressure dynamics. The pressures generated by the reduced model along with the flow rate determine a pressure boundary condition that is imposed in the detailed model. Complete details are provided in prior work.[48,61] Pressure is imposed on $\partial\Omega$ by prescribing a combination of normal traction and no-slip tangential velocity boundary conditions.[48,61–63] We assume spatially uniform pressure profiles at the junctions between the detailed and reduced models. The numerical implementation of these boundary conditions allows for either inflow or outflow.[48,61,63] Regions of $\partial\Omega$ that are outside of these junctions are set to have zero pressure conditions, which are specified by a combination of homogeneous normal traction and no-slip tangential velocity boundary conditions.[62,63] This zero-pressure boundary condition is taken as the "ground" pressure within the model and enables fluid to flow into or out from the computational domain to accommodate changes in the volume of the overall heart model. Although not considered here, it would be straightforward to instead impose a time-dependent external pressure that accounts for the intrathoracic pressure dynamics associated with breathing.

As detailed in Methods Section *Cardiac biomechanics models*, the biomechanical response of the heart, its valves, and the nearby great vessels are described using hyperelastic constitutive models that are formulated using elastic energy functionals $\mathcal{W}$ of invariants and pseudo-invariants of the right Cauchy-Green strain, $\mathbb{C} = \mathbb{F}^{\mathsf{T}}\mathbb{F}$, in which $\mathbb{F} = \partial\chi/\partial\mathbf{X}$ is the deformation gradient tensor and $J = \det(\mathbb{F})$ is the Jacobian determinant. Although the continuum formulation of the IFED method generates exactly incompressible deformations, for which $J \equiv 1$, this property is generally lost when the continuous equations are discretized because of both spatial and temporal discretization effects.[59] To mitigate these errors, we have found that it is beneficial to adopt a nearly incompressible material formulation.[59] To do so, it is convenient to introduce the so-called modified Cauchy-Green strain, $\overline{\mathbb{C}} = \overline{\mathbb{F}}^{\mathsf{T}}\overline{\mathbb{F}}$ with $\overline{\mathbb{F}} = J^{-\frac{1}{3}}\mathbb{F}$. Notice that $\det(\overline{\mathbb{F}}) = 1$, so $\overline{\mathbb{C}}$ encodes only deviatoric deformations but not dilatational motions. We denote by $\overline{\mathcal{W}}$ elastic energies that use invariants of $\overline{\mathbb{C}}$, i.e., in terms of the modified invariant $\overline{I}_1 = \mathrm{tr}(\overline{\mathbb{C}})$ instead of $I_1 = \mathrm{tr}(\mathbb{C})$. We do not use modified pseudo-invariants, because doing so can result in non-physical deformations.[64] In addition, we introduce a volumetric energy,

$$\mathcal{U}(J) = \beta_{\mathrm{s}}(J\log(J) - J + 1), \tag{S18}$$

which penalizes changes in volume, in which β_s is the numerical bulk modulus.[59] The numerical bulk modulus β_s is set to 4.0e4 kPa, and the same value is used throughout the entire structure. We use $\overline{\mathcal{W}}$ and $\mathcal{U}$ to evaluate the first Piola-Kirchhoff elastic stress via

$$\mathbb{P} = \frac{\partial \overline{\mathcal{W}}}{\partial \mathbb{F}} + \frac{\partial \mathcal{U}}{\partial \mathbb{F}}. \tag{S19}$$

See Vadala-Roth et al.[59] for further details.

Our computations use an adaptive staggered-grid discretization of the incompressible Navier-Stokes equations detailed by Griffith[48] and a finite element description of Lagrangian equations describing the deformation the immersed structures and the resulting force generation.[57] The interaction equations, Eqs. (S13) and (S15), are discretized using an efficient nodal coupling scheme,[56] and we replace the singular delta function with a regularized three-dimensional delta function $\delta_h(\mathbf{x})$ in tensor-product form,[53–55,65]

$$\delta_h(\mathbf{x}) = \prod_{i=1}^{3} \delta_h(x_i), \tag{S20}$$

in which the one-dimensional regularized delta function is defined in terms of a basic kernel function,

$$\delta_h(x) = \frac{1}{h}\varphi\left(\frac{x}{h}\right). \tag{S21}$$

In our simulations, we set $\varphi(r)$ to be a three-point B-spline kernel that was found to provide excellent accuracy and robustness compared to other commonly used choices.[58] The function $\Psi_w(\mathbf{x})$ used for both distributing the source strengths and sampling the pressures is similarly defined in tensor-product form, except that we use a cosine kernel.[55,66] We treat w as a fixed length scale that, in our basic discretization, is four times the Cartesian grid spacing on the finest level of the locally refined grid. The same fixed value of w is also used in grid convergence tests reported in Supplementary Results Section *Grid convergence studies*. The locally refined Cartesian grid is comprised of two nested grid levels with a refinement ratio of four between levels, and it provides a fine-grid spatial resolution of 0.86 mm. We use second-order centered differences for the Eulerian divergence, gradient, and Laplace operators along with a high-resolution upwind scheme for the convective terms.[48] We use standard P^2 (quadratic) tetrahedral elements to describe the structure. The Eulerian and Lagrangian variables are coupled using an explicit midpoint method,[48] and the incompressible Navier-Stokes equations are discretized in time using a semi-implicit scheme that uses the Crank-Nicolson method for the viscous terms and the second-order Adams-Bashforth scheme for the convective terms. We use a time-step size of 2.69 μs, which was chosen to be as large as possible while avoiding volumetric instability in the structure.

Flow rate, chamber volume, and pressure

We identify valve annuli upstream of each of the four heart valves, and we construct surface meshes where blood velocities are sampled to evaluate volumetric flow rates through each valve. The flow rate is captured every 100 time steps. Flow volumes associated with each valve are obtained by integrating the flow rate via the trapezoidal rule. After the volume changes are computed, the

flow rate data and volume contributions are down sampled to every 0.01 s for plotting output. The volumes that pass through the aortic valve and mitral valve are used in conjunction with the initial volume of the ventricle to compute the evolving left ventricular volume. The initial volume of the left ventricle was determined by extracting a surface mesh of the left ventricular endocardium that was then capped by the mitral and aortic valve annular meshes. The mesh manipulation and volume computation were done using Meshmixer (Autodesk, San Rafael, CA). The measured pressures in the atria and ventricles correspond to the mean of the pressure field calculated within small regions contained within the chambers' endocardial surfaces. This region dynamically moves with the deformation of the heart tissue. It is sampled by a cosine kernel centered at the current position of the endocardium centroid and with a radius that is four times the finest Cartesian grid spacing. While there is some spatial variation in the pressure field, the calculated pressure is relatively insensitive to the location and size of this region. Pressure data are down sampled to every 0.01 s and smoothed using a three point moving average by the `smooth` function in MATLAB (MathWorks, Natick, MA).

Parameter summary

This section briefly summarizes the origins of all parameters used in the model. We specifically identify the parameters that were kept fixed throughout the present study along with those that were adjusted to produce the physiological results reported herein. Methods Section *Cardiac biomechanics models* provides further details, including specific approaches to model calibration, as well as the specific parameter values.

Most of the model parameters were held fixed and were based on prior characteristic studies. These include the parameters for the tissue constitutive equations, which were derived from stress-strain tests performed on dissected human tissue samples. For the ventricular myocardium and papillary muscles, we used the parameter set from the literature stress-strain study as the constitutive equation matched our desired anisotropoic model.[27] For the four valves[31] and the chordae,[33,34] we performed our own parameter fits. We performed our own fits because the study that provided the stress-strain data for the valves fit the data to a two-dimensional biaxial Fung-type model rather than a three-dimensional constitutive equation, and because the studies that provided the data for the chordae did not provide fits to any constitutive equations. The specific protocol for fitting the valve and chordae parameters is included in Methods Section *Material characterization*. The model parameters for the great vessels were chosen to limit gross deformations throughout the cycle, and the bulk modulus was chosen to be large enough for volumetric preservation while being small enough to allow for a reasonable time step size. Similarly, the parameters for the pericardial tethering were chosen to limit oscillations of the epicardial surface during contraction. The dynamic viscosity and the density for the fluid are based on literature measurements for both at the organ scale.[47] The inductance and resistance parameters for the atrial fluid sources were based on previous simulation work and were chosen for numerical stability, and the pulmonary venous pressure was determined to provide sufficient cardiac output.[52]

Many of the remaining model parameters were modified within the model calibration process. Most prominently, the compliance and the peripheral resistance of the systemic circulation were determined to provide physiologic systemic diastolic and systolic pressure values at the aortic boundary, as outlined in Methods Section *Blood and circulation*, whereas the characteristic resistance of the systemic circulation was obtained from the literature.[49] In addition, the timing and magnitude of the chamber contractions were not derived from the literature, with the exception of the delay

between atrial and ventricular contraction.[43] The stroke volume of the ventricle was the main determinant of the timings and magnitudes of the remaining contraction parameters. Valvular flow rate statistics, including the mitral E/A wave ratio and the peak aortic valve flow rate, were also considered.

Supplementary Results

Left ventricular end-diastolic pressure-volume relationship

To provide a justification of the material parameters of the left ventricle, we generated the Klotz pressure-volume curve as detailed by Klotz et al.[67] to test the passive elastic response of the model heart. To do so, we removed the pericardial tethering from the ventricular epicardium, applied loads of 5, 10, 15, 20, 25, and 30 mmHg to the endocardial surface of the left ventricle, and recorded the resulting chamber volume. The Klotz relation relies on a normalized volume $\tilde{v}(p)$ defined by

$$\tilde{v}(p) = \frac{v(p) - v_0}{v_{30} - v_0}, \tag{S22}$$

in which $v(p)$ is the volume (mL) of the left ventricle at pressure p (mmHg), v_{30} is the volume of the left ventricle at 30 mmHg, and v_0 is the volume of the left ventricle at 0 mmHg. Figure S2 shows the Klotz curve data generated by our model. We obtain good agreement to the reference curve, with a root mean squared error of 1.63 mmHg. This error is comparable to the root mean squared error of 2.99±1.72 mmHg reported by Klotz et al. for in vivo human data.[67]

Left ventricular volume dynamics

Figure S3 shows the time series data for the left ventricular volume that were used to generate the pressure-volume relation. The pressure data are shown in the main body of the text, but volume waveforms are much less commonly obtained in vivo. As mentioned in Methods Section *Flow rate, chamber volume, and pressure*, the left ventricular volume was estimated using the fluxes through the mitral and aortic valves along with an initial volume that was determined using numerical quadrature.

Grid convergence studies

Figure S6 shows time series data for the pressure, flow rate, and ejection volume through the whole heart's aortic valve model mounted in the aortic valve test section model developed by Lee et al.[58,68] Because the heart model results discussed in this study are predominantly concerned with pressures, flow rates, and volumes, we focused our convergence study on these results. We consider the aortic valve because it is the smallest valve in the model, so the number of Cartesian grid cells spanning the aortic valve is the lowest. The coarsest grid considered in this convergence study preserves the grid resolution across the aorta in the whole heart model, and we chose to consider two levels of refinement, at 1.5x and 2x the baseline resolution used in the whole heart model. To replicate the conditions generated by the model, we used the pressure and flow rate from the model upstream of the aortic valve to generate an appropriate upstream boundary condition. The same Windkessel

model that provides afterload for the left side of the heart in the whole heart model was applied at the downstream outlet of the aortic valve test section.

Figure S6 demonstrates good qualitative agreement across the considered grid resolutions. We further quantified the maximum flow rates, maximum pressures, and ejection volumes. With grid refinement the maximum downstream pressure, corresponding the pressure in the left ventricular outflow tract, decreased from 135.1 mmHg to 132.8 mmHg and 132.1 mmHg for 1.5x and 2x refinement, respectively, which corresponds to a 2.3% and 0.53% difference for the coarser meshes when the finest level is used as the reference. The maximum downstream pressure, corresponding the pressure in the ascending aorta, increased from 131.5 mmHg to 133.3 mmHg and 133.7 mmHg for 1.5x and 2x refinement, respectively, which corresponds to a 1.6% and 0.31% difference for the coarser meshes when the finest level is used as the reference. The maximum flow rate through aortic valve decreased from 464.4 mL/s to 478.9 mL/s and 485.7 mL/s for 1.5x and 2x refinement, respectively, which corresponds to a 4.4% and 1.4% difference for the coarser meshes when the finest level is used as the reference. Finally, the ejection volume through the aortic valve increased from 62.14 mL to 63.41 mL and 63.91 mL for 1.5x and 2x refinement, respectively, which corresponds to a 2.8% and 0.8% difference for the coarser meshes when the finest level is used as the reference.

We also consider convergence of the flow fields generated across the valve tester at the aortic valve orifice, as shown in Figure S7. We chose to sample the velocity orthogonal to the imaging slice during the ramp up to peak ejection, at timepoints when the flow rate is approximately half of the maximum value and when the model reaches peak ejection flow rate through the valve. We see good qualitative agreement across the resolutions considered herein.

This analysis was extended to the mitral valve in the whole heart model using the same steps in resolution. We considered the time series data through the first diastolic phase. Figure S8 shows the mitral flow rate, left ventricular pressure, and left ventricular volume. These data show that the maximum flow rate slightly decreases with increased resolution, from 165.5 mL/s at the original resolution to 160.9 mL/s and 152.6 mL/s for the 1.5x and 2x refinement cases, respectively. We conducted a orthogonal velocity comparison for the velocity across the mitral valve during the ramp up to peak flow rate and during the peak flow rate, and the results in this study are included as Figure S9. As with the aortic valve tester, we see good qualitative agreement with the orthogonal flow velocity profiles.

While the local velocity results are encouraging, the bulk of the whole heart results are concerned explicitly with more gross aspects of the cardiac cycle, such as flow rates through the valve orifices and averaged pressures at the centroids of the chambers, rather than highly localized velocity and pressure measures. Further convergence investigations of local velocities and pressures for the whole heart would benefit from cycle averaging studies, especially when considering the convergence of the flow features presented in Figure 8, because the in vivo data provided from 4D flow MRI are cycle averaged. A comprehensive convergence study for localized velocities and pressures in the whole heart model would require further rigorous and extensive experimental work, including simulations and comparisons to in vitro and in vivo 4D flow MRI data, to meet the expectations of the FDA standard.[69] Such additional validation would clearly be necessary for this platform to be used in medical device design or clinical decision making.

Supplementary Discussion

Analysis of the Frank-Starling response

Here we provide additional analysis of the Frank-Starling response generated by the model heart. Figure 9(d) demonstrates that the peak ventricular pressure varies by approximately 10 mmHg for the range of pulmonary venous pressures considered, and we see a small increase in the end-systolic volume of 1.3 mL between the 10 mmHg load and 14 mmHg load case. Ventricular function curves (Figure 9(c)) have a well-known plateau, in which stroke volume becomes insensitive to further increases in preload,[70–72] and these results suggest that we have nearly reached this regime in the present study. Figure 9(d) also shows that the left ventricle pressurizes more quickly under larger loads, but the pressure loss during isovolumetric relaxation happens at the same pace, suggesting that the increased atrial pressure is driving the initial pressure gain in the left ventricle.

The mitral flow rate waveform is also clearly impacted by the changes in atrial loading pressure, as seen in Figure 9(e). The magnitudes of the E and A waves both increase with increased preload. Considering the E wave, this suggests that diastolic ventricular loading is not only a function of ventricular relaxation but is also driven by atrial load. These results support the interpretation that our atrium is properly functioning as a reservoir.[73] Interestingly, with faster left ventricular loading and higher pressures, flow through the aortic valve begins earlier but does not reach the same peak magnitude, as illustrated in Figure 9(f).

An observable difference between the pressure-volume relation from the simulated Frank-Starling response (Figure 9(a)) and the in vivo results (Figure 6)(c)) is the smaller changes to the end-systolic pressure and volume from the model. In the in vivo pressure-volume relation, the reduction in vena cava occlusion produces larger end-systolic pressures and volumes along with the increasing end-diastolic volume in a quasi-linear relationship that captures the end-systolic pressure-volume relationship. From our experiment, we also see increases in the maximum left ventricular pressure, reflective of the increased afterload, with increased end-diastolic volume (Figure 9(a) and (d)); however, the end-systolic volume does not substantially change with increased atrial load. We believe this results from the comparative difference in the preload changes, in which the end-diastolic volume changes by approximately 10 mL in the in vivo experiment from cycle to cycle whereas the simulation changes are approximately 2 mL. This phenomenon of variable end-diastolic loading changes leading to distinct end-systolic pressure-volume relations is observed in vivo.[70] The contractility associated with active strain contraction model could also have an effect on the end-systolic pressure because, if the active strain model confers a high degree of contractility, the end-systolic volume increase would be reduced.[74,75] Comparisons of myocardial contractility across various approaches to modeling active contraction (e.g., active strain vs. active stress) do not appear to have been reported for fluid-structure interaction models of the heart. Indeed, it is known that it is not possible to convert an arbitrary active strain contraction model to an active stress formulation (or vice versa),[26] and so we would anticipate that different approaches to modeling contraction would generate different end-systolic pressure-volume relationships. A simulation study comparing the Frank-Starling response across various active strain and active stress contraction models could help to determine if one approach to modeling contraction is in better agreement with in vivo data, but, to our knowledge, such a comparison has yet to be performed.

Supplementary Videos

Video S1: Chamber and valve dynamics along with blood flow velocity magnitudes shown on a plane approximately bisecting the mitral and aortic valves.

Video S2: Chamber and valve dynamics along with blood pressures shown on a plane approximately bisecting the mitral and aortic valves.

Video S3: Chamber and valve dynamics along with out-of-plane vorticity shown on a plane approximately bisecting the mitral and aortic valves.

Video S4: Detailed aortic valve kinematics along with three-dimensional blood flow velocity fields.

Video S5: Detailed mitral valve kinematics along with three-dimensional blood flow velocity fields.

	EDV (mL)	ESV (mL)	SV (mL)	EF	CO ($L \cdot min^{-1}$)
**Model**	127.30	51.73	75.57	0.59	4.5
**Reference**	110-120[50]	40-50[50]	70[50](39.1–98.5[76])	0.6[50]	~5[50](3.09-9.0[76])

Table S5: Performance metrics extracted from the left ventricular pressure-volume loop. ESV = end-systolic volume, EDV = end-diastolic volume, SV = stroke volume, EF = ejection fraction, and CO = cardiac output. CO is directly computed from the stroke volume and the heart rate (60 BPM). Model outputs are in excellent agreement with reference physiological values.[50] Parenthetical ranges are included to illustrate clinically measured population variability.[76]

	Reservoir (%)	Conduit (%)	Pump (%)
**Model**	34.4	47.4	18.1
**Reference**[73]	37.5	36.5	26

Table S6: Performance metrics extracted from the left atrial pressure-volume relationship. The reservoir, conduit, and pump volume fractions for the left atria are reported as percentages of the ventricular stroke volume generated by the model (75.57 mL) and compared to reference values for healthy human subjects.[73]

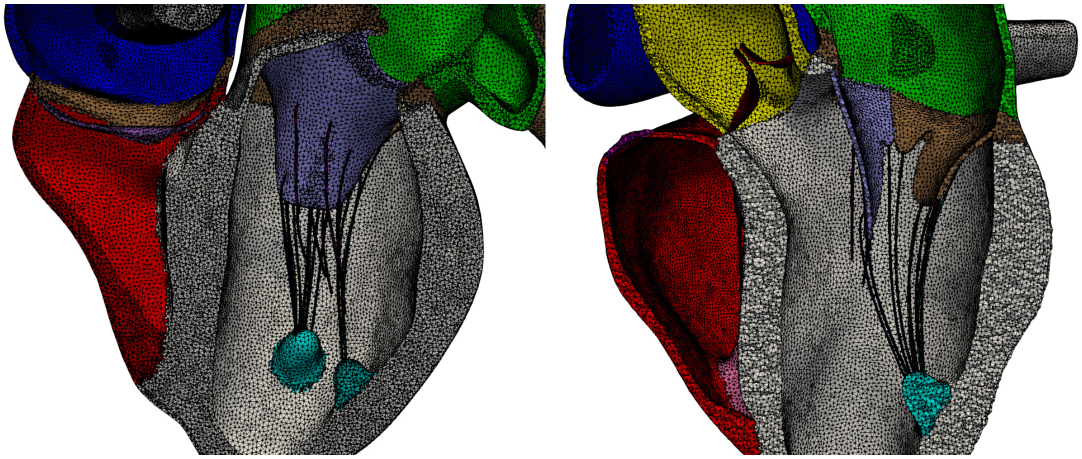

Fig. S1: A visualization of the heart mesh with a focus on the left side and mitral valve apparatus. The mesh contains approximately 2.4 million tetrahedral elements with an average diameter of 1.17 mm. The colors denote different subdomains within the mesh.

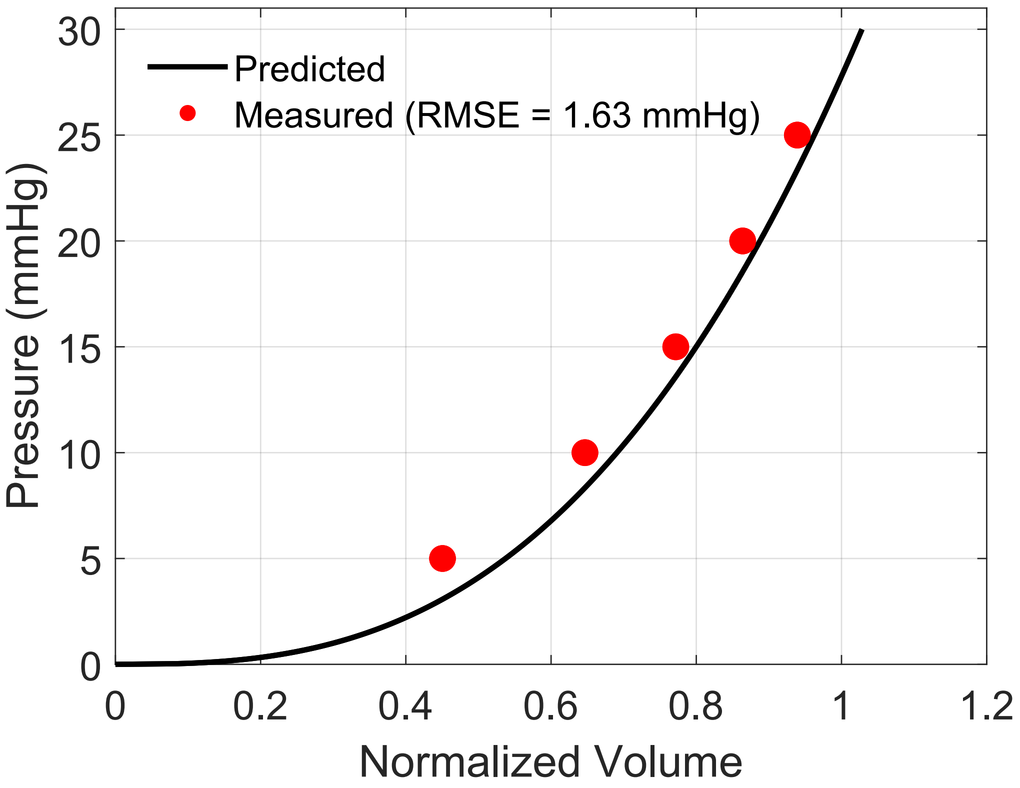

Fig. S2: The Klotz curve generated from our model, which was used to test the passive material parameters within the left ventricle.[67] Pressures of 5, 10, 15, 20, 25, and 30 mmHg were applied to the left ventricular endocardial surface with pericardial tethering removed from the epicardial surfaces. The resultant left ventricular volumes were 115.8, 124.3, 129.8, 133.8, 137.0, and 139.7 mL, respectively, with an initial left ventricular volume of 96.3 mL. Compared to the predicted curve estimated (black curve) by Klotz et al., the root mean squared error of the observed data (red dots) is 1.63 mmHg, which is well within to the observed in vivo human data range of 2.99±1.72 mmHg.[67]

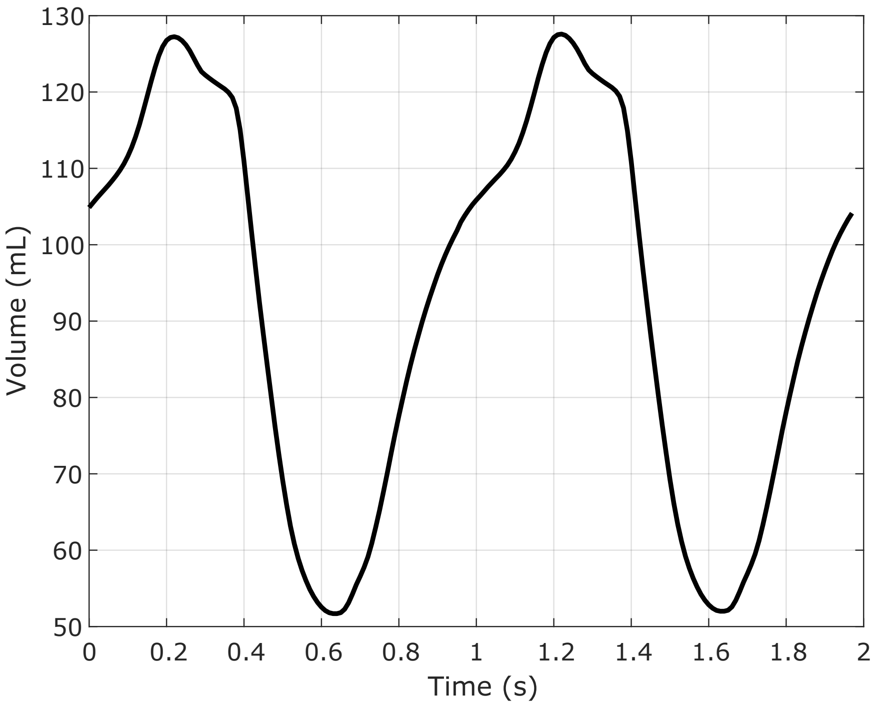

Fig. S3: The total left ventricular volume during two successive cycles.

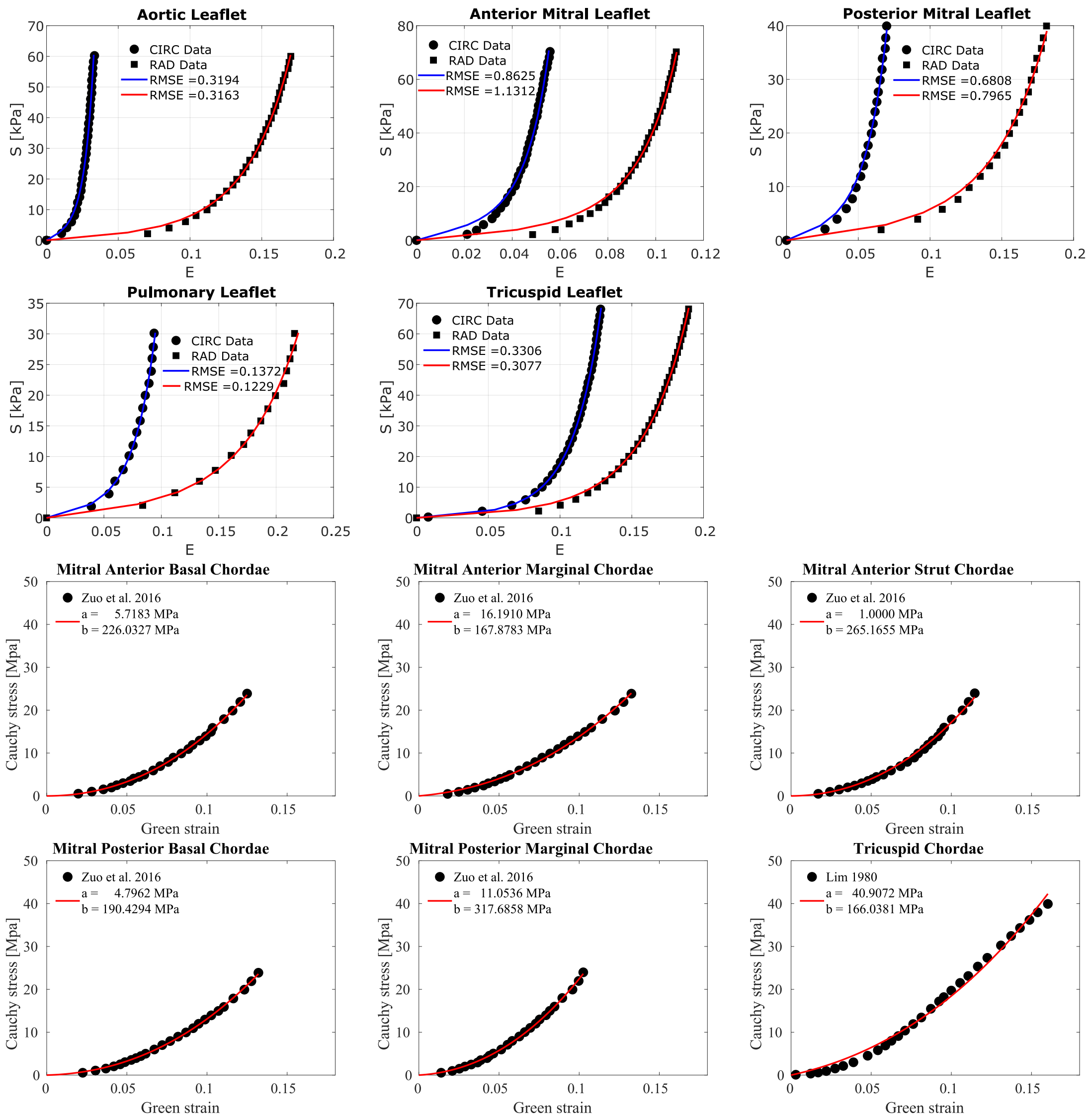

Fig. S4: Parameters for the valve material models were derived from the results of biaxial stress-strain tests along the radial and circumferential directions executed by Pham et al.,[31] and fit to the modified Holzapfel-Gasser-Ogden model by Murdock et al.,[30] Eq. (S3). Data from mitral[33] and tricuspid[34] chordae stress-strain tests were fit to a generic nonlinear spring material model, Eq. (S4). Digitized data are shown as the black circles and squares for circumferential and radial fiber directions, respectively, for the valves. The fits for the radial and circumferential data are shown as the red and blue lines, respectively, along with the corresponding root mean squared errors (RMSE) for the valve fits. The digitized data for the chordae are shown as black circles, and the fits are shown as the red curves with the resultant parameters listed.

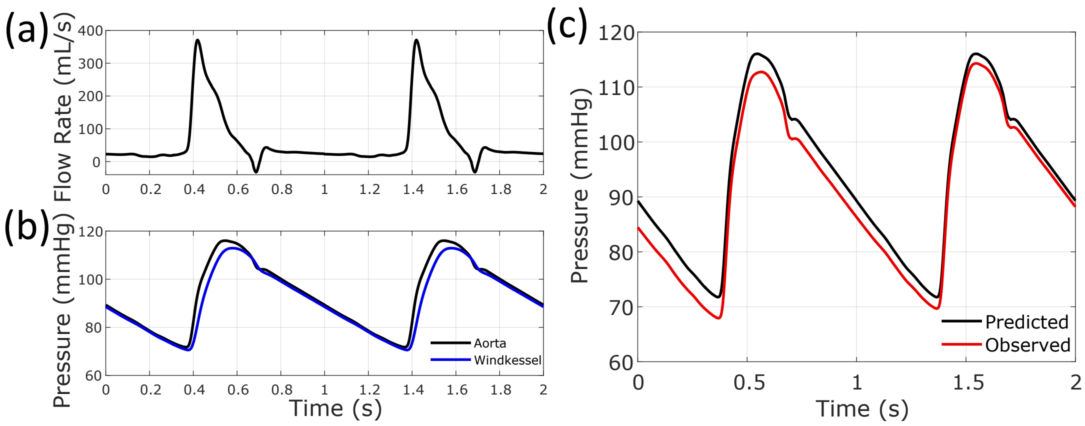

Fig. S5: Workflow for tuning the Windkessel parameters for the aorta, where the Windkessel pressure is a surrogate for the systemic pressure downstream of the ascending aorta. (a) The tuning of the Windkessel parameters starts with extracting the flow rate waveforms from the top of the aorta. (b) The flow rate is then used to choose the compliance and peripheral resistance that lead to appropriate systolic and diastolic pressures in the aorta. (c) The model is then run with these parameters and the observed pressure values are compared against the predicted pressure waves. Over successive cycles, the observed pressure approaches the pressure wave predicted by the specified Windkessel model parameters.

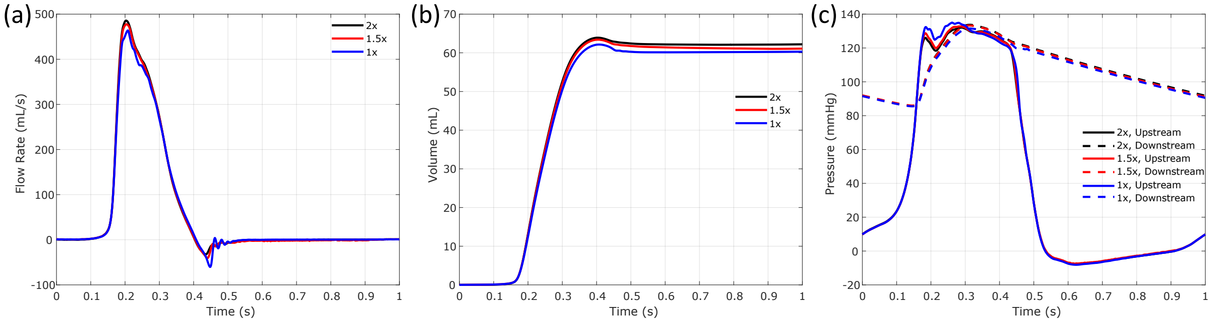

Fig. S6: Convergence results for (a) the flow rate through, (b) the ejection volume through, and (a) the pressures upstream and downstream of the aortic valve geometry in a static aorta test section for the resolution of the model, and 1.5x and 2x Eulerian domain grid refinements.

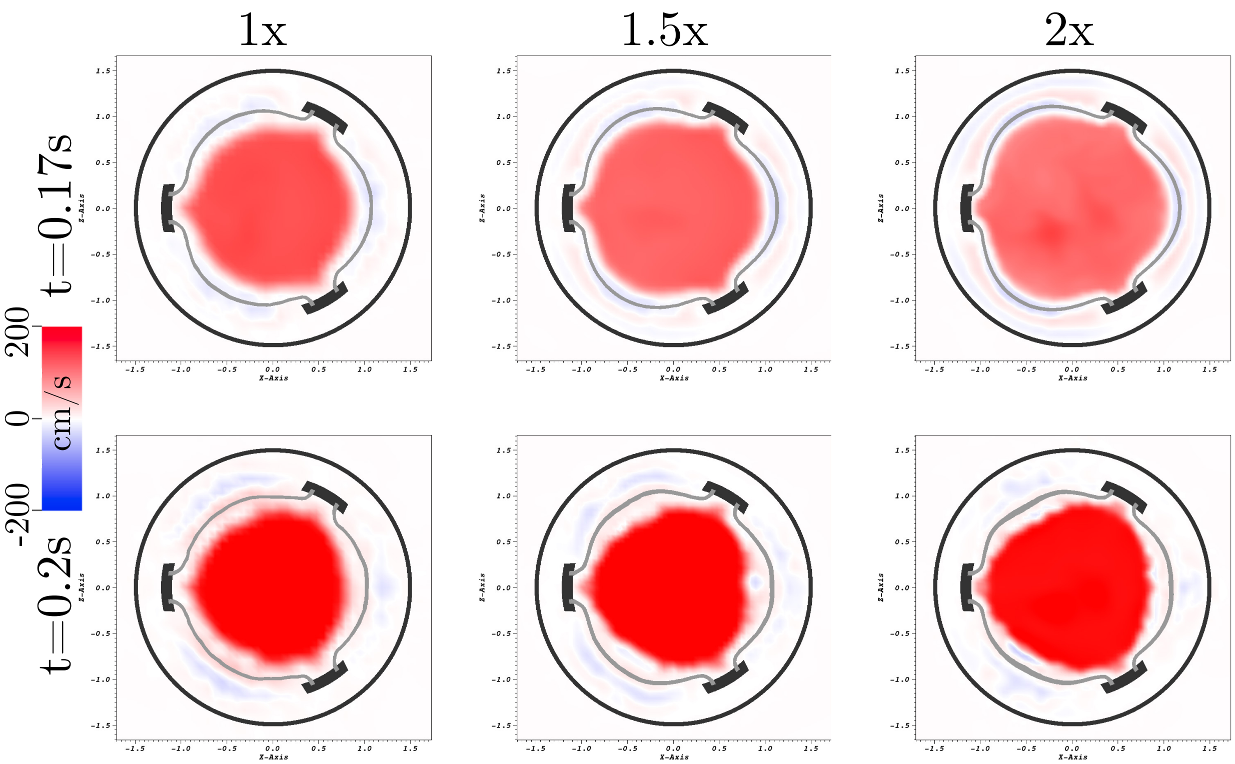

Fig. S7: Convergence results for the orthogonal velocity measurements across the aortic valve geometry in the static aorta test section for the resolution of the model, and 1.5x and 2x Eulerian domain grid refinements. The timepoints align with the data presented in Figure S6, and were chosen as approximately half the maximum flow rate on the initial ramp up (0.17s) and the maximum flow rate (0.2s). The slice is oriented from downstream of the aortic valve looking upstream, and positive velocity is flow passing out of the plane, or flow passing through the aortic valve from upstream to downstream.

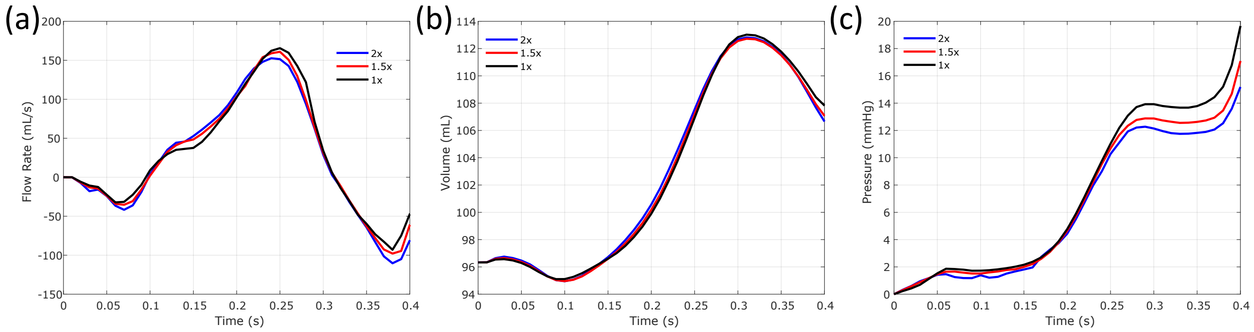

Fig. S8: Convergence results for the (a) flow rate through the mitral valve, (b) the left ventricular volume, and (c) the left ventricular pressure in the whole heart setup during diastole the resolution of the model, and 1.5x and 2x Eulerian domain grid refinements.

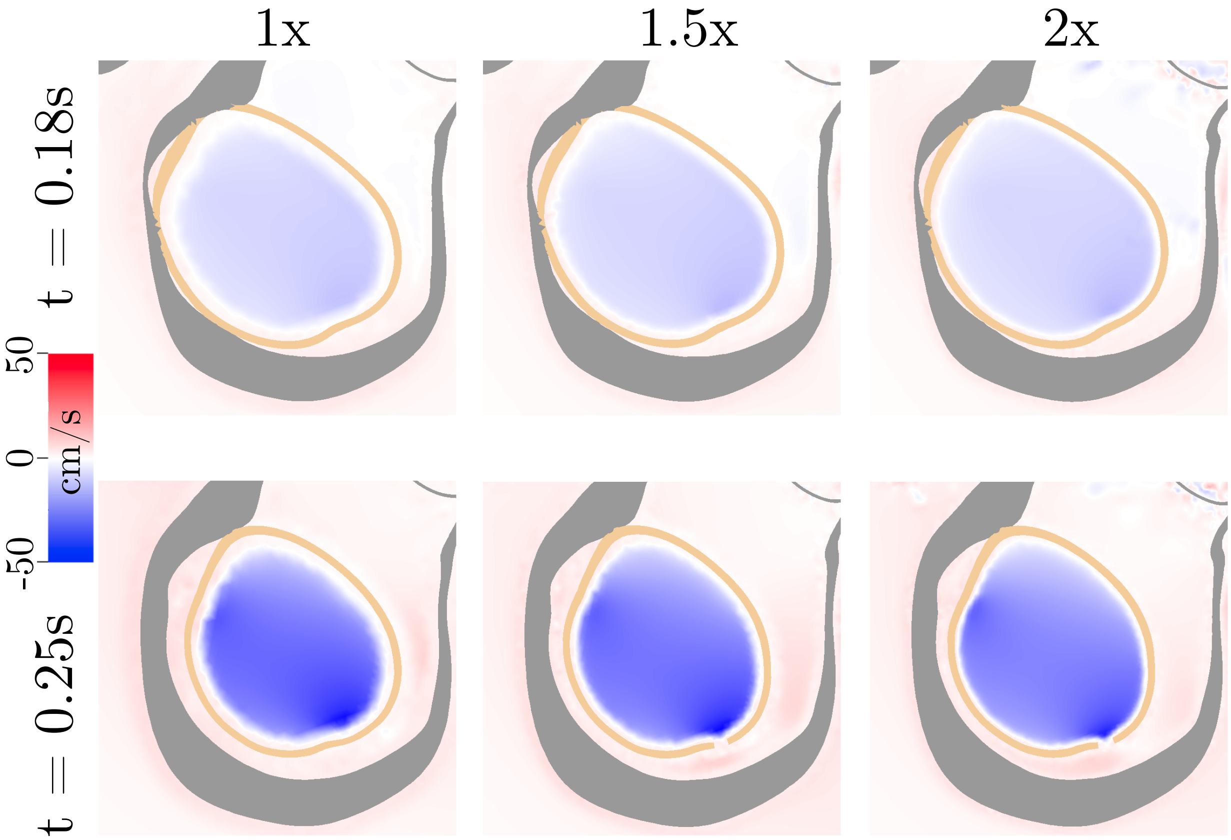

Fig. S9: Convergence results for the orthogonal velocity measurements across the mitral valve in the whole heart model during the first diastolic phase for the original resolution of the model, and 1.5x and 2x Eulerian domain grid refinements. The timepoints align with the data presented in Figure S8, and were chosen as approximately half the maximum flow rate on the initial ramp up (0.18s) and the maximum flow rate (0.25s). The slice is oriented looking from the left atrium into the left ventricle through the mitral valve annulus with the negative velocity indicating flow into the plane, or from the left atrium into the left ventricle.

Supplementary References

[1] Segars WP, Mahesh M, Beck TJ, Frey EC, Tsui BM (2008) Realistic CT simulation using the 4D XCAT phantom. *Med Phys* 35(8):3800–3808.

[2] Segars WP, Sturgeon G, Mendonca S, Grimes J, Tsui BM (2010) 4D XCAT phantom for multimodality imaging research. *Med Phys* 37(9):4902–4915.

[3] Hasan A, et al. (2017) Image-based immersed boundary model of the aortic root. *Med Eng Phys* 47:72–84.

[4] Reul H, et al. (1990) The geometry of the aortic root in health, at valve disease and after valve replacement. *J Biomech* 23(2):181–191.

[5] Swanson WM, Clark RE (1974) Dimensions and geometric relationships of the human aortic valve as a function of pressure. *Circ Res* 35(6):871–882.

[6] Clark RE, Finke EH (1974) Scanning and light microscopy of human aortic leaflets in stressed and relaxed states. *J Thorac Cardiovasc Surg* 67(5):792–804.

[7] Khalighi AH, Drach A, Gorman RC, Gorman JH, Sacks MS (2018) Multi-resolution geometric modeling of the mitral heart valve leaflets. *Biomech Model Mechanobiol* 17(2):351–366.

[8] Lim KH, Yeo JH, Duran CM (2005) Three-dimensional asymmetrical modeling of the mitral valve: a finite element study with dynamic boundaries. *J Heart Valve Dis* 14(3):386–392.

[9] Prot V, Skallerud B (2009) Nonlinear solid finite element analysis of mitral valves with heterogeneous leaflet layers. *Comput Mech* 43:353–368.

[10] Lama P, Tamang BK, Kulkarni J (2016) Morphometry and aberrant morphology of the adult human tricuspid valve leaflets. *Anat Sci Int* 91(2):143–150.

[11] Skwarek M, Grzybiak M, Kosiński A, Hreczecha J (2004) Notes on the morphology of the tricuspid valve in the adult human heart. *Folia Morphol (Warsz)* 63(3):319–324.

[12] Khalighi AH, et al. (2019) Development of a functionally equivalent model of the mitral valve chordae tendinae through topology optimization. *Ann Biomed Eng* 47(1):60–74.

[13] Hu Y, Schneider T, Wang B, Zorin D, Panozzo D (2020) Fast tetrahedral meshing in the wild. *ACM Trans Graph* 39(4):117:1–117:18.

[14] Bigi A, et al. (1982) Structure and orientation of collagen fibres in human mitral valve. *Int J Biol Macromol* 4(7):387–392.

[15] Driessen NJ, Bouten CV, Baaijens FP (2005) A structural constitutive model for collagenous cardiovascular tissues incorporating the angular fiber distribution. *J Biomech Eng* 127(3):494–503.

[16] Wong J, Kuhl E (2014) Generating fibre orientation maps in human heart models using Poisson interpolation. *Comput Methods Biomech Biomed Engin* 17(11):1217–1226.

[17] Rossi S, Lassila T, Ruiz-Baier R, Sequeira A, Quarteroni A (2014) Thermodynamically consistent orthotropic activation model capturing ventricular systolic wall thickening in cardiac electromechanics. *Eur J Mech A Solids* 48(1):129–142.

[18] Arts T, Costa KD, Covell JW, McCulloch AD (2001) Relating myocardial laminar architecture to shear strain and muscle fiber orientation. *Am J Physiol Heart Circ Physiol* 280(5):H2222–H2229.

[19] Nielsen PM, Le Grice IJ, Smaill BH, Hunter PJ (1991) Mathematical model of geometry and fibrous structure of the heart. *Am J Physiol* 260(4):H1365–H1378.

[20] Streeter DD, Hanna WT (1973) Engineering mechanics for successive states in canine left ventricular myocardium. II. Fiber angle and sarcomere length. *Circ Res* 33(6):656–664.

[21] Holzapfel GA, Ogden RW (2009) Constitutive modelling of passive myocardium: a structurally based framework for material characterization. *Philos Trans A Math Phys Eng Sci* 367(1902):3445–3475.

[22] Ho SY, Cabrera JA, Sánchez-Quintana D (2012) Left atrial anatomy revisited. *Circ Arrhythm Electrophysiol* 5(1):220–228.

[23] Ferrer A, et al. (2015) Detailed anatomical and electrophysiological models of human atria and torso for the simulation of atrial activation. *PLoS One* 10(11):e0141573.

[24] Krueger MW, et al. (2011) Modeling atrial fiber orientation in patient-specific geometries: A semi-automatic rule-based approach in *Functional Imaging and Modeling of the Heart*, eds. Metaxas D, Axel L. (Springer Berlin Heidelberg, Berlin, Heidelberg), pp. 223–232.

[25] Rossi S, et al. (2022) Rule-based definition of muscle bundles in patient-specific models of the left atrium. *Front Physiol* 13:912947.

[26] Ambrosi D, Pezzuto S (2012) Active stress vs. active strain in mechanobiology: Constitutive issues. *J Elast* 107(2):199–212.

[27] Gültekin O, Sommer G, Holzapfel GA (2016) An orthotropic viscoelastic model for the passive myocardium: continuum basis and numerical treatment. *Comput Methods Biomech Biomed Engin* 19(15):1647–1664.

[28] Augustin CM, et al. (2019) The impact of wall thickness and curvature on wall stress in patient-specific electromechanical models of the left atrium. *Biomech Model Mechanobiol* 19(3):1015–1034.

[29] Holzapfel GA, Gasser TC, Ogden RW (2000) A new constitutive framework for arterial wall mechanics and a comparative study of material models. *J Elast* 61(1):1573–2681.

[30] Murdock K, Martin C, Sun W (2018) Characterization of mechanical properties of pericardium tissue using planar biaxial tension and flexural deformation. *J Mech Behav Biomed Mater* 77:148–156.

[31] Pham T, Sulejmani F, Shin E, Wang D, Sun W (2017) Quantification and comparison of the mechanical properties of four human cardiac valves. *Acta Biomater* 54:345–355.

[32] Gasser TC, Ogden RW, Holzapfel GA (2006) Hyperelastic modelling of arterial layers with distributed collagen fibre orientations. *J R Soc Interface* 3(6):15–35.

[33] Zuo K, et al. (2016) Characterization of biomechanical properties of aged human and ovine mitral valve chordae tendineae. *J Mechan Behav Biomed Mater* 62:607–618.

[34] Lim KO (1980) Mechanical properties and ultrastructure of normal human tricuspid valve chordae tendineae. *Jpn J Physiol* 30(3):455–464.

[35] Rohatgi A (2022) Webplotdigitizer: Version 4.6.

[36] Ambrosi D, Arioli G, Nobile F, Quarteroni A (2011) Electromechanical coupling in cardiac dynamics: the active strain approach. *SIAM J on Appl Math* 71(2):605–621.

[37] Stålhand J, Klarbring A, Holzapfel GA (2011) A mechanochemical 3d continuum model for smooth muscle contraction under finite strains. *Journal of theoretical biology* 268(1):120–130.

[38] Rossi S, Ruiz-Baier R, Pavarino LF, Quarteroni A (2012) Orthotropic active strain models for the numerical simulation of cardiac biomechanics. *Int J Numer Method Biomed Eng* 28(6-7):761–788.

[39] Göktepe S, Menzel A, Kuhl E (2014) The generalized hill model: A kinematic approach towards active muscle contraction. *Journal of the Mechanics and Physics of Solids* 72:20–39.

[40] Quarteroni A, Lassila T, Rossi S, Ruiz-Baier R (2017) Integrated heart – coupling multiscale and multiphysics models for the simulation of the cardiac function. *Comput Methods Appl Mech Eng* 314:345–407.

[41] Barbarotta L, Rossi S, Dedè L, Quarteroni A (2018) A transmurally heterogeneous orthotropic activation model for ventricular contraction and its numerical validation. *Int J Numer Method Biomed Eng* 34(12):e3137.

[42] Fröhlich J, et al. (2023) Numerical evaluation of elasto-mechanical and visco-elastic electro-mechanical models of the human heart. *GAMM-Mitteilungen* 46(3-4):e202370010.

[43] Houthuizen P, Bracke FA, van Gelder BM (2011) Atrioventricular and interventricular delay optimization in cardiac resynchronization therapy: physiological principles and overview of available methods. *Heart Fail Rev* 16(3):263–276.

[44] Murgo JP, Westerhof N, Giolma JP, Altobelli SA (1980) Aortic input impedance in normal man: relationship to pressure wave forms. *Circulation* 62(1):105–116.

[45] Pfaller MR, et al. (2019) The importance of the pericardium for cardiac biomechanics: from physiology to computational modeling. *Biomech Model Mechanobiol* 18(2):503–529.

[46] Peskin CS, McQueen DM (1996) Fluid dynamics of the heart and its valves in *Case Studies in Mathematical Modeling: Ecology, Physiology, and Cell Biology*, eds. Othmer HG, Adler FR, Lewis MA, Dallon JC. (Prentice-Hall, Englewood Cliffs, NJ, USA), pp. 309–337.

[47] Brindise MC, Busse MM, Vlachos PP (2018) Density and viscosity matched Newtonian and non-Newtonian blood-analog solutions with PDMS refractive index. *Exp Fluids* 59(11):173.

[48] Griffith BE (2012) Immersed boundary model of aortic heart valve dynamics with physiological driving and loading conditions. *Int J Numer Method Biomed Eng* 28(3):317–345.

[49] Stergiopulos N, Westerhof BE, Westerhof N (1999) Total arterial inertance as the fourth element of the windkessel model. *Am J Physiol* 276(1):H81–H88.

[50] Hall JE (2016) *Guyton and Hall Textbook of Medical Physiology*. (Elsevier, Philadelphia), Thirteenth edition.

[51] Naeije R (2013) Physiology of the pulmonary circulation and the right heart. *Curr Hypertens Rep* 15(6):623–631.

[52] Griffith BE (2005) Ph.D. thesis (New York University).

[53] Peskin CS (2002) The immersed boundary method. *Acta Numer* 11:479–517.

[54] Peskin CS (1972) Flow patterns around heart valves: A numerical method. *J Comput Phys* 10(2):252–271.

[55] Peskin CS (1977) Numerical analysis of blood flow in the heart. *J Comput Phys* 25(2):220–252.

[56] Wells D, Vadala-Roth B, Lee JH, Griffith BE (2023) A nodal immersed finite element-finite difference method. *J Comput Phys* 477:111890.

[57] Griffith BE, Luo X (2017) Hybrid finite difference/finite element immersed boundary method. *Int J Numer Method Biomed Eng* 33(12):e2888.

[58] Lee JH, Griffith BE (2022) On the Lagrangian-Eulerian coupling in the immersed finite element/difference method. *J Comput Phys* 457:111042.

[59] Vadala-Roth B, Acharya S, Patankar NA, Rossi S, Griffith BE (2020) Stabilization approaches for the hyperelastic immersed boundary method for problems of large-deformation incompressible elasticity. *Comput Methods Appl Mech Eng* 365:112978.

[60] Boffi D, Gastaldi L, Heltai L, Peskin CS (2008) On the hyper-elastic formulation of the immersed boundary method. *Comput Methods Appl Mech Eng* 197(25-28):2210–2231.

[61] Lee J, et al. (2020) Fluid-structure interaction models of bioprosthetic heart valve dynamics in an experimental pulse duplicator. *Ann Biomed Eng* 48(5):1475–1490.

[62] Gresho PM, Sani RL (1998) *Incompressible Flow and the Finite Element Method: Advection-Diffusion and Isothermal Laminar Flow*. (John Wiley & Sons).

[63] Griffith BE (2009) An accurate and efficient method for the incompressible Navier-Stokes equations using the projection method as a preconditioner. *J Comput Phys* 228(20):7565–7595.

[64] Sansour C (2008) On the physical assumptions underlying the volumetric-isochoric split and the case of anisotropy. *Front Physiol* 27(1):28–39.

[65] Tornberg AK, Engquist B (2004) Numerical approximations of singular source terms in differential equations. *J Comput Phys* 200(2):462–488.

[66] Engquist B, Tornberg AK, Tsai R (2005) Discretization of Dirac delta functions in level set methods. *J Comput Phys* 207(1):28–51.

[67] Klotz S, et al. (2006) Single-beat estimation of end-diastolic pressure-volume relationship: a novel method with potential for noninvasive application. *Am J Physiol Heart Circ Physiol* 291(1):H403–H412.

[68] Lee J, et al. (2021) Bioprosthetic aortic valve diameter and thickness are directly related to leaflet fluttering: results from a combined experimental and computational modeling study. *JTCVS Open* 6:60–81.

[69] Center for Devices and Radiological Health (2023) Assessing the credibility of computational modeling and simulation in medical device submissions in *Guidance for Industry and Food and Drug Administration Staff*. (Food and Drug Administration, Rockville, MD).

[70] Jacob R, Dierberger B, Kissling G (1992) Functional significance of the Frank-Starling mechanism under physiological and pathophysiological conditions. *Eur Heart J* 13 Suppl E:7–14.

[71] Kosta S, Dauby PC (2021) Frank-Starling mechanism, fluid responsiveness, and length-dependent activation: Unravelling the multiscale behaviors with an in silico analysis. *PLoS Comput Biol* 17(10):e1009469.

[72] Han JC, Loiselle D, Taberner A, Tran K (2021) Re-visiting the Frank-Starling nexus. *Prog Biophys Mol Biol* 159:10–21.

[73] Pagel PS, et al. (2003) Mechanical function of the left atrium: new insights based on analysis of pressure-volume relations and doppler echocardiography. *Anesthesiology* 98(4):975–994.

[74] Sagawa K (1981) The end-systolic pressure-volume relation of the ventricle: definition, modifications and clinical use. *Circulation* 63(6).

[75] Antonini-Canterin F, et al. (2013) The ventricular-arterial coupling: From basic pathophysiology to clinical application in the echocardiography laboratory. *J Cardiovasc Echogr* 23(4):91–95.

[76] Cattermole GN, et al. (2017) The normal ranges of cardiovascular parameters measured using the ultrasonic cardiac output monitor. *Physiol Rep* 5(6):e13195.
